# A comprehensive overview of strategies to improve blueberry fruit quality

**DOI:** 10.3389/fpls.2026.1833867

**Published:** 2026-05-11

**Authors:** Tiago Lopes, Ana Paula Silva, António Augusto Vicente, Berta Gonçalves

**Affiliations:** 1 Centre for the Research and Technology of Agro-Environmental and Biological Sciences (CITAB), University of Trás-os-Montes e Alto Douro (UTAD), Vila Real, Portugal; 2 Institute for Innovation, Capacity Building and Sustainability of Agri-Food Production (Inov4Agro), University of Trás-os-Montes e Alto Douro, Vila Real, Portugal; 3 CEB - Centre of Biological Engineering, University of Minho, Braga, Portugal; 4 LABBELS - Associate Laboratory, Braga/Guimarães, Portugal

**Keywords:** biostimulants, blueberry quality, edible coatings, phenolic compounds, postharvest strategies, preharvest strategies

## Abstract

Blueberry (*Vaccinium* spp.) is one of the most economically and nutritionally important crops worldwide due to its sensory attributes and high levels of bioactive compounds, which contribute to human health. Fruit quality is defined by a combination of biometric, chromatic, organoleptic, and textural aspects, as well as biochemical properties such as soluble sugar levels, organic acids, polyphenols, vitamin C content, and antioxidant capacity. In recent years, numerous sustainable preharvest methods have been developed to improve plant physiological development, increase nutrient-use efficiency, enhance stress tolerance, modulate primary and secondary metabolic pathways, and improve fruit yield and quality. Among these methods are the application of biostimulants and calcium. However, because blueberries are highly perishable, adopting postharvest strategies that delay ageing, maintain firmness, and preserve biochemical components is essential to reduce postharvest losses. Low-temperature storage, controlled and modified atmosphere conditions, non-thermal and light-based technologies, innovative packaging systems, and the use of biopolymer-based edible coatings are among the most commonly employed techniques. Therefore, combining pre- and postharvest strategies offers a practical way to ensure fruit quality, extend shelf life, and meet consumer demands.

## 1 Introduction

The growing popularity of blueberries is attributed to their unique sensory profile and their renowned status as a ‘superfruit’ (Sater et al., 2021). Over the past 20 years, blueberry production has substantially grown, making it one of the fastest-growing fruit crops in the world. International Blueberry Organization (IBO) data show that world production in 2024 was approximately 2.17 million metric tons, with China, Peru, and the United States being the largest fresh blueberry producers (Brazelton et al., 2025). This fruit is an excellent source of bioactive compounds, such as flavonoids, phenolic acids, anthocyanins, tannins, and vitamins (Wang et al., 2017a; Rokayya et al., 2021), providing numerous health benefits, including antioxidant, anti-inflammatory and antitumour activity, neuroprotective effects, improved vision, and cardiovascular protection (Miraghajani et al., 2020; Kalt et al., 2020; Duan et al., 2022; Ashique et al., 2024). Consequently, blueberries are now available in a variety of formats beyond fresh, including frozen products, juices, and snacks (Calabuig-Jiménez et al., 2022).

To translate these nutritional advantages into market value, it is essential to maintain and enhance fruit characteristics throughout the entire production process and subsequent supply chain. Contemporary consumers want their blueberries to be fresh, sweet, firm, and defect-free (Ehlenfeldt and Martin, 2002; Gallardo et al., 2018), but fruit quality is influenced by several factors, such as soil and climatic conditions, genotype, geographic region, stage of ripeness, and storage conditions (Cardeñosa et al., 2016; Correia et al., 2016; Shi et al., 2023a). Therefore, achieving high quality at harvest is essential to ensure optimal postharvest performance. This requires appropriate selection of blueberry cultivars, balancing vegetative and reproductive growth, and harvesting at optimal maturity (Forney, 2009). The adoption of innovative, sustainable pre- and postharvest strategies to address these quality and shelf-life challenges has been considered to improve blueberry production outcomes. The preharvest application of biostimulants represents a promising strategy for increasing not only fruit yield but also organoleptic quality, nutritional content, and biological potential by enhancing plant physiological efficiency (Gonçalves et al., 2025). At the same time, postharvest edible coatings are essential for prolonging shelf life and maintaining commercial attributes. These coatings, typically formulated with biopolymers like polysaccharides, proteins, and lipids, act as semipermeable barriers that delay senescence and microbial deterioration and can also be functionalized with bioactive compounds to confer additional properties (Flores-López et al., 2016; Vieira et al., 2016).

In this context, the main objective of this review is to analyse and summarize critical factors related to blueberry quality. Specifically, it seeks to: (i) characterise the main quality attributes of blueberry fruit; (ii) evaluate the potential of preharvest application of biostimulants and calcium (Ca) as a sustainable strategy to improve plant physiological performance and overall fruit quality; (iii) review the role of edible coatings applied postharvest as an effective technology for preserving quality, extending marketability, and preventing losses, in line with market demands for safe and sustainable solutions. This integrated analysis aims to provide a comprehensive, up-to-date perspective for producers, researchers, and industry to develop more efficient practices that deliver a high-value product to consumers, thereby maximizing the nutritional and commercial value of blueberries.

## 2 Blueberry quality factors

### 2.1 Biometric properties

Blueberry biometric parameters are key determinants for genetic improvement and market value. Fruit size, ease of detachment from the peduncle, and reduced stem scar size are among the characteristics most valued by producers, breeders, and consumers (Hancock et al., 2008; Retamales and Hancock, 2018). Among these, fruit size and calibre are highly relevant commercial criteria, used as indirect quality indicators. Fruit weight varies significantly among blueberry genotypes in accordance with cellular determinants, with rabbiteye cultivars producing smaller fruits (1.16 g) compared to southern highbush (SHB) cultivars with a higher mean fruit weight (2.14 g) than northern highbush (NHB) (1.92 g) (Gündüz et al., 2015). The stem scar is also an important quality parameter, since, despite occupying a small fraction of the blueberry’s surface, it has higher transpiration rates than the cuticle. As such, fruits with larger scars lose more water and generally have lower firmness during storage, thereby compromising their quality and shelf life (Moggia et al., 2017a). These qualitative aspects, as well as the concentration and properties of the bloom, reflect differences in fruit dehydration rates and vary according to genotype, environment, and cultural practices employed (Moggia and Lobos, 2023).

It is also important to note that blueberry size is correlated with other quality attributes. For example, Gündüz et al. (2015) observed negative associations between fruit weight and total soluble solids (TSS), total phenolic content (TPC), and antioxidant capacity (AC), suggesting that an improvement in size may, on the other hand, lead to the production of more acidic fruits with lower AC. There is therefore a need for integrated approaches to the biometric analysis of blueberries, and it is essential to consider the physical dimensions, anatomical bases, and physiological and functional implications for fruit quality.

### 2.2 Colour, maturity, and texture

Colour, maturity and texture are interconnected quality parameters that change concurrently during blueberry ripening, shaped by the interaction of genotype, environment, and physiological mechanisms. Understanding these attributes in an integrated manner is essential for defining optimal harvest windows, selecting high-performance cultivars, and designing effective quality management strategies.

Colour is one of the most important indicators of blueberry ripeness and quality (Júnior et al., 2025) and is a complex characteristic influenced by various physical and chemical factors. The light blue colour of this fruit is a result of the structure and quantity of epicuticular wax (bloom), and the concentration of anthocyanins present in the epidermis (Retamales and Hancock, 2018). Additionally, the final blue/purple colouration of fruit is caused by anthocyanin accumulation and chlorophyll breakdown, which are controlled by genetic factors such as the miR156-VcSPL12 module (Li et al., 2020b). As ripening progresses, this translates into a consistent decline in L*, a*, and b* values compared with unripe fruits (Hwang et al., 2020). However, this progression is not uniform, with asynchrony between individual berries, even within the same bunch or on the same branch (Doi et al., 2021). Furthermore, the spatial and temporal pattern of colour development varies across blueberry types. For example, NHB blueberries exhibit a more uniform colour change, while SHB cultivars display a mosaic of colours throughout ripening. Rabbiteye blueberries, in turn, show distinct ripening patterns (Zapien-Macias et al., 2025).

At the biochemical level, fruit ripening involves a major adjustment in metabolism. In this process, the concentration of sugars (e.g., glucose and fructose), TSS, pH, and anthocyanins increases, while titratable acidity (TA) and the concentration of organic acids and TPC tend to decrease, leading to an increase in the maturity index (MI), based on the ratio between TSS and TA, which significantly affects flavour and perceived quality and can be used as an integrative harvest indicator (Gilbert et al., 2015; Aires et al., 2017; Retamales and Hancock, 2018; Hwang et al., 2020). This is also accompanied by hormonal changes: auxins (IAA) and gibberellins (GA_3_), which are predominant in the early stages, decrease, while abscisic acid (ABA) reaches maximum levels in the final stages of ripening, showing a strong positive correlation with the accumulation of anthocyanins and sugars (Aires et al., 2017). On the other hand, the overall energy status (e.g., ATP, ADP, and AMP) and AC of this fruit tend to decrease as fruit ripening progresses (Aires et al., 2017; Shi et al., 2023a), a finding with implications for postharvest storage potential.

Blueberry texture is largely controlled by cell wall composition and integrity. In general, mechanical firmness measurements quantify the resistance of fruit tissue to compression, penetration, or deformation, whose values depend largely on the integrity of the cell membranes. In turn, the chemical composition of pectin, which bind plant cells, is fundamental to this aspect (Chiabrando et al., 2009; Paniagua et al., 2014). Cultivars with a crunchier pulp texture have higher amounts of hemicelluloses (e.g., arabinans, heteromannans, xyloglucans) and cellulose (Trandel-Hayse et al., 2023) and may have a longer harvest interval (Moggia et al., 2016). As ripening advances, the progressive enzymatic loosening of the cell wall reduces structural integrity, leading to softening that is further promoted by ABA-mediated upregulation of cell wall-degrading enzymes and the consequent collapse of middle mesocarp cells (Liu et al., 2022). A study of blueberries harvested at two different ripeness stages (i.e., fully blue and 6 days later) showed that firmness can decrease by up to 24% between these stages, depending on the year and cultivar, highlighting the importance of frequent harvesting (Lobos et al., 2018). Thus, a high MI is associated with decreased fruit firmness, increasing the probability of internal browning after impact and during storage (Moggia et al., 2017b; Hwang et al., 2020). These attributes can therefore help to estimate the ideal harvest date and storage potential. Indeed, an increase in the amount of soft fruit during transport tends to lead to rejections in the destination market in years with adverse environmental conditions, such as high temperatures close to harvest (Moggia et al., 2017a). The concentration of epicuticular wax, such as bloom, on berries increases throughout fruit development (Yang et al., 2020), adding another layer of complexity to fruit quality attributes and influencing not only visual appearance but also water loss and susceptibility to physical damage. Although environmental factors can influence fruit texture, genetics are the main controller of this parameter (Vilela et al., 2016; Moggia et al., 2022; Ferrão et al., 2024). However, fruit firmness is a characteristic that requires careful analysis. Consumers define ideal or unpleasant texture based on their own perceptions, which may not be affected by objective measurements (Giongo et al., 2022) and the adoption of non-destructive, accurate, and robust techniques for texture measurement is essential to reliably assess fruit quality. These attributes are most effectively comprehended as an integrated system rather than as separate quality traits. The significant cultivar-by-environment interaction observed (Vilela et al., 2016; Cappai et al., 2018) demonstrates the importance of multi-site research that goes beyond single-season evaluations in order to develop prediction models useful for business decision-making.

### 2.3 Sensory attributes

Consumers usually evaluate fruit quality based on appearance, flavour, texture, and scent (Moggia et al., 2022). As a result, understanding consumer preferences is crucial in meeting the needs of that specific market. The taste of blueberries is influenced by parameters such as fruit pH, fructose, and volatile organic compounds (VOCs), which affect sourness and sweetness. In general, sourness negatively affects overall liking of the fruit, whereas sweetness, taste, and texture qualities generally have a positive impact (Gilbert et al., 2015). Blueberries with aromatic notes of linalool, p-cymene, D-limonene, and myrtenal terpenes are preferred by consumers due to their appealing aromatic perception (Ferrão et al., 2022). The intensity of blueberry odour, sweetness, and taste correlate with soluble sugar content, while succulence is associated with better texture. Bitter taste and taste intensity are linked to odour and flavour perception (Vilela et al., 2016). Although there is a strong link between MI and overall liking, sourness, and sweetness, the individual measures of SSC and TA accounted for a greater proportion of the variance in sensory qualities (Gilbert et al., 2015). In terms of customer acceptability and willingness to pay (WTP), the three most important factors are flavour liking, flavour intensity, and sweetness intensity. The amount of sugar in blueberries also correlates positively with customers’ WTP. The relationship between the concentration of organic acids in blueberries and WTP is complex; some levels of concentration will improve flavour perception and WTP, while excessive levels will negatively affect both. Furthermore, customer preferences for firmer blueberries are evident, as higher WTP is typically associated with greater blueberry firmness (Canales et al., 2024). However, seed presence is the most significant negative attribute in overall texture evaluation (Gilbert et al., 2014).

Blueberry aroma is derived from VOCs produced during fruit ripening. Although several techniques are used to extract these compounds, over 120 VOCs were found in highbush blueberries, over 80 in rabbiteye, and approximately 40 in lowbush blueberries, with highbush cultivars displaying the most diverse profiles (Sater et al., 2020). When detected by our olfactory and taste receptors, blueberry flavour is produced by a blend of VOCs, sugars, and acids (Dias et al., 2023). In highbush cultivars, the major aromatic compounds are linalool, (Z)-3-hexen-1-ol, geraniol, α-terpineol, and 1-octen-3-one (Forney et al., 2022). A study of six highbush cultivars identified 58 glycosidically bound volatile compounds: 29 derived from lipid oxidation, 23 terpenoids, 5 from the shikimic acid pathway, and 1 norisoprenoid (Pico et al., 2023). The NHB ‘Draper’ and ‘Duke’ cultivars had, respectively, 73.1% and 70.7% of lipid oxidation derivatives, 25% and 26% of terpenoids, and 1.9% and 3.3% of shikimic acid derivatives. Using the gas chromatography-olfactometry technique, 42 organic volatile compounds were identified in highbush cultivars, of which 48% were aldehydes, 26% were monoterpenoids, and 3% were esters. On the other hand, ethyl propanoate, methyl 2-methylbutanoate, methyl 3-methylbutanoate, ethyl 2-methylbutanoate, and ethyl 3-methylbutanoate were the major aromatic compounds found in lowbush blueberries, which exhibit sweeter and fruiter profiles (Forney et al., 2022). Rabbiteye cultivars are characterised by higher terpene content than lowbush varieties (Sater et al., 2020). Analysis of five rabbiteye cultivars revealed 53 volatile compounds, the most significant of which were terpenoids, including linalool, alcohols, aldehydes, and esters. Among these, the most relevant were linalool, hexanal, hexyl acetate, limonene, methyl 3-methylbutanoate, 1,8-cineole, (Z)-3-hexenal, (Z)-3-hexenyl acetate, a-terpineol and (E)-2-hexanal (Beaulieu et al., 2014). Among VOCs, terpenoids, esters, ketones, alcohols, and aldehydes can interfere with and explain changes in blueberry aroma (Farneti et al., 2017).

As blueberries ripen, significant changes occur in flavour, aroma, and volatile compound composition, with riper fruits exhibiting a more intense flavour profile and taste (Farneti et al., 2017; Cheng et al., 2020; Shi et al., 2023a). The composition of VOCs varies not only with maturity but also among blueberry cultivars (Sater et al., 2020) and is influenced by climatic conditions, which affect colour, sweetness, acidity, aroma, astringency, crispness, and earthy flavour (Vilela et al., 2016). The aroma of blueberries can undergo significant changes during cold storage, with a rapid decline in terpenoid content and varying levels of ethyl acetate. However, cold storage can slow the loss of aromatic compounds compared to ambient temperature storage (Yan et al., 2020).

### 2.4 Biochemical composition and antioxidant capacity

#### 2.4.1 Sugars and organic acids

Sugars and organic acids significantly influence the sensory perception of blueberries, particularly flavour, aroma, and consumer acceptance, highlighting the importance of balancing these compounds (Bett‐Garber et al., 2015; Gilbert et al., 2015; Mengist et al., 2021). The predominant soluble sugars in highbush blueberries are fructose (64.06–304.52 mg g^^-^1^ DW), glucose (13.86–57.36 mg g^^-^1^ DW) and galactose (11.40–58.52 mg g^^-^1^ DW). High levels of citric acid (13.34–87.81 mg g^^-^1^ DW), quinic acid (2.86–14.78 mg g^^-^1^ DW), and malic acid (1.02–7.21 mg g^^-^1^ DW) have also been reported in blueberries, although the values vary depending on the ripeness stage, environmental conditions, cultural practices and the specific cultivar (Correia et al., 2016; Ordóñez-Díaz et al., 2020; Zhang et al., 2020; Oh et al., 2025). According to Wang et al. (2019), citric acid represents over 90% of the total acids quantified in several diploid and tetraploid *V*. *corymbosum* clones. On the other hand, rabbiteye blueberries have higher concentrations of succinic acid (50%) and malic acid (34%) (Ehlenfeldt et al., 1994). The accumulation of these metabolites is greatly influenced by the stage of fruit development, driven by interconnected metabolic pathways such as sucrose hydrolysis, glycolysis, the tricarboxylic acid (TCA) cycle, and the shikimate pathway (Li et al., 2020a). Through ripening, sugar content increases progressively, while organic acid concentration decreases, particularly citric acid. For example, between immature and fully ripe blueberries of ‘Bluecrop’, ‘Goldtraube’, ‘Ozarkblue’, and ‘Duke’ cultivars, there was a two- to four-fold reduction in citric acid content, with an opposite trend observed between sugars and organic acids (Aires et al., 2017). These changes may be related to increased cell membrane permeability during ripening, leading to the mobilization and metabolization of organic acids (Yang and Hinner, 2015).

#### 2.4.2 Bioactive compounds and antioxidant capacity

Blueberries present high levels of bioactive compounds, including polyphenols, anthocyanins, and vitamin C. These metabolites, due to their antioxidant and anti-inflammatory activities and potential chemopreventive effects, confer several properties that promote human well-being and health, although their concentration is subject to significant variation across genotypes, soil conditions, and climate (Bunea et al., 2013; Correia et al., 2016; Działo et al., 2016; Das et al., 2022; Lopes et al., 2026). Specifically, phenolic compounds represent a diverse group of molecules that share an aromatic ring, with 8,000 distinct structures identified (Działo et al., 2016). In red fruits, including blueberries, the main identified classes are phenolic acids and anthocyanins, while other types, such as flavonols, are also present, albeit at lower concentrations (Cosme et al., 2022). The accumulation of these compounds in fruits follows a spatial and temporal pattern throughout ripening (Dare et al., 2022; Albert et al., 2023), initially in the epidermal vacuoles (Li et al., 2019b). In comparison to rabbiteye blueberries (1512 µg gallic acid equivalents (GAE) g^-1^ FW), NHB (1914 µg GAE g^-1^ FW) and SHB (1887 µg GAE g^-1^ FW) cultivars had a higher mean of TPC, according to a study conducted by Gündüz et al. (2015) on blueberries grown in Oregon in 2012.

Among phenolic compounds, flavonoids represent a major and functionally relevant subclass. Such compounds have a basic structure (C6-C3-C6) and are grouped into six main subclasses: flavonols, flavones, flavanones, flavan-3-ols, isoflavones, and anthocyanidins (Działo et al., 2016). Several flavonoid glycosides have been identified in blueberries, some of which are specific to certain cultivars, such as the pentosides, glucosides, and galactosides of isorhamnetin, syringetin, and laricetin (Vrhovsek et al., 2012). Anthocyanins are natural pigments belonging to the flavonoid group responsible for the blue, red and purple colour of blueberries, which can represent up to 60% of the TPC in ripe fruits (Bendokas et al., 2020; Kalt et al., 2020). The main anthocyanidins present in highbush blueberries are derivatives of cyanidin, delphinidin, malvidin, peonidin, and petunidin (Kim et al., 2025). Among these, delphinidin and malvidin derivatives are the most common, making up 31.4% and 29.1% of total anthocyanins, respectively (Wang et al., 2019; Jung et al., 2021), with particular emphasis on delphinidin-3-*O*-galactoside, which is generally the most abundant (Aires et al., 2017; Lopes et al., 2026). The anthocyanin content of fruits tends to increase as they ripen and its consumption has been linked to a lower risk of mortality, primarily due to a reduction in cardiovascular diseases (Grosso et al., 2017; Shi et al., 2023a), and to its high antioxidant, anti-inflammatory, and insulin-sensitising properties (Yang et al., 2022). The regulation of flavonoid and anthocyanin biosynthesis in blueberries is mediated by MYB transcription factors, which control the expression of structural genes encoding key biosynthetic enzymes via the MBW complex (MYB-bHLH-WD40). In this fruit, specific *VcMYB* factors (i.e., *VcMYB397*, *VcMYB193*, *VcMYB391*, and *VcMYB369*) are differentially expressed during fruit development and ripening, acting as key components of the anthocyanin production pathway. Additionally, *VcMYBPA1.1* shows peak expression during both early and late developmental stages, coinciding with proanthocyanidin biosynthesis and anthocyanin accumulation, respectively, with many *VcMYB*s showing preferential transcript accumulation in the fruit skin, consistent with the deposition of anthocyanins in this species (Wang et al., 2023a). TPC and AC tend to correlate positively with total TSS, confirming the possibility of combining a high content of flavour-related compounds (e.g., sugars) with health-promoting compounds with high antioxidant activity. On the other hand, acidic compounds such as caffeic acid, chlorogenic acid, and vitamin C correlate with TA and contribute to the fruit’s perceived acidity (Mazzoni et al., 2020).

Vitamin C is a water-soluble antioxidant with a high capacity to neutralise free radicals and reactive oxygen species (ROS) and constitutes a key component in the chemical profile of blueberries (Wang and Jiao, 2000; Gomes-Rochette et al., 2016; Gęgotek and Skrzydlewska, 2022). The vitamin C concentration of highbush blueberries is generally high, with considerable variability between cultivars and species within the *Vaccinium* genus (Prior et al., 1998). This compound is also influenced by the ripeness of the fruit, exposure to stress, and environmental factors such as light intensity, photoperiod, and temperature (Lee and Kader, 2000). During ripening, for example, there was a significant increase in ascorbic acid concentration in ‘Bluecrop’ (+ 281%), ‘Duke’ (+ 192%), and ‘Goldtraube’ (+ 156%) blueberry cultivars (Aires et al., 2017). According to Cosme et al. (2022), the vitamin C content of blueberries can range from 0.10 to 1.00 mg g-1 on a fresh weight (FW) basis. Specifically, Vitamin C levels varied between 0.13 and 1.05 mg g^−1^ of dry weight (DW) among ‘Duke’ and ‘Draper’ cultivars grown in Portugal over two years (Lopes et al., 2026).

The AC of blueberries is primarily attributed to their rich composition of phenolic compounds, particularly anthocyanins, flavonoids, phenolic acids, and vitamin C, which have a high capacity to neutralise free radicals and chelate transition metals (Moyer et al., 2002; Li et al., 2017; Sun et al., 2018; Zhou et al., 2020). Among the factors influencing AC, fruit maturity plays a central role. As ripening progresses, the concentration of phenolic compounds increases, leading to correspondingly higher AC values that reflect the intensification of oxidative defence mechanisms through enhanced antioxidant enzyme activity (Hwang et al., 2020; Shi et al., 2023a). On the other hand, unripe fruits may exhibit greater inhibitory activity against acetylcholinesterase and butyrylcholinesterase, whose inhibition elevates acetylcholine levels at cholinergic synapses, a mechanism with recognised relevance in the management of neurodegenerative conditions such as Alzheimer’s disease (Hwang et al., 2020).Due to differences in phenolic profiles, AC can also vary by approximately two to threefold across cultivars (Kraujalytė et al., 2015; Wang et al., 2017a). Furthermore, anthocyanin concentration was positively correlated with AC assessed by the FRAP assay, which in turn showed a positive association with soluble sugar content (Gündüz et al., 2015). By observing this correlation, it is possible to develop blueberry cultivars that combine high AC values with favourable sensory attributes. In addition to classic AC, blueberry extracts have also demonstrated the ability to reduce nitric oxide (NO^•^) radicals and inhibit the enzyme α-glucosidase, with values higher than those of positive controls such as ascorbic acid and acarbose, respectively (Gonçalves et al., 2023). Beyond species and cultivar differences, the AC of blueberries also varies considerably with environmental factors and the analytical method used. For example, Gündüz et al. (2015) found that blueberries cultivated in Oregon had average FRAP values of 61.40 µmol TE g^-1^ FW for NHB, 57.80 µmol TE g^-1^ FW for rabbiteye, and 56.40 µmol TE g^-1^ FW for SHB. Lopes et al. (2026) discovered similar significant variability for ‘Duke’ and ‘Draper’ NHB cultivars grown in Portugal. The AC of these cultivars ranged from 86.03 to 190.03 µmol TE g^-1^ DW using ABTS, 55.61 to 130.29 µmol TE g^-1^ DW using CUPRAC, and 60.20 to 146.65 µmol TE g^-1^ DW using DPPH. According to Cosme et al. (2022), ORAC values for various blueberry cultivars ranged from 10.3 to 51.9 µmol g^-1^ FW. Generally, high light intensity, UV radiation, and warmer climates stimulate the phenylpropanoid pathway, promoting secondary metabolite accumulation in more stress-tolerant plants and the biosynthesis of flavonoids and anthocyanins as photoprotective responses, thereby increasing AC (Zoratti et al., 2014; Mazzoni et al., 2020). The flavonoid biosynthesis pathway is also one of the most consistently enriched in drought-responsive genes, indicating that water deficit can upregulate phenolic compound production (Feng et al., 2024).

## 3 Preharvest strategies to enhance blueberry production and fruit quality

### 3.1 Application of biostimulants

A plant biostimulant is defined as any substance or microorganism applied to plants to enhance nutrition efficiency, abiotic stress tolerance, and crop quality traits, regardless of its nutrient content (Du Jardin, 2015). As metabolic enhancers, biostimulants accelerate plant development at low application rates through the novel or emergent properties they contain (Yakhin et al., 2017). Because of their minimal environmental impact and their role in reducing the use of pesticides and fertilizers, these products are seen as an alternative to conventional agronomic methods (Calvo et al., 2014; Yakhin et al., 2017; Regni et al., 2021), promoting the activity of beneficial microorganisms and interacting with signalling molecules synthesized by plants, improving water and mineral utilization by plants, and promoting tolerance to biotic and abiotic stresses, flowering, fruit set, and crop growth (Brown and Saa, 2015; Colla et al., 2015; Van Oosten et al., 2017; Ali et al., 2021). The impact of biostimulant application in conventional and organic cultivation systems has been widely documented. For instance, its application in open−field conditions provides an average yield increase of 17.9%, with more positive effects observed in regions with arid climates and in sandy soils with low organic matter content, salinity issues, and nutritional deficiencies (Li et al., 2022). According to Du Jardin (2015), the main categories of biostimulants are seaweed extracts, fulvic and humic acids, protein hydrolysates and other N-containing compounds, beneficial microorganisms, chitosan and other biopolymers and inorganic compounds. This author also considers amino acids such as glutamate (GLU) and phytohormones such as 6−benzylaminopurine (6−BAP) to be biostimulants.

#### 3.1.1 Humic and fulvic acids

Humic acids (HA) and fulvic acids (FA) are humic substances with high chemical complexity, resulting from the chemical and biochemical transformation of plant and animal organic matter produced during the humification process (Canellas et al., 2015; Guo et al., 2019). This process involves the slow degradation and structural reorganization of organic materials arising from the breakdown of plant- and microbe-derived residues through biological, chemical, and physical mechanisms, giving rise to heterogeneous mixtures with no defined chemical formula, rich in carboxylic, phenolic, quinone, and hydroxyl functional groups (De Melo et al., 2016; Yang et al., 2021). Globally, humic substances account for approximately 25% of total organic carbon (Weber et al., 2018).

The distinction between FA and HA lies in their solubility characteristics. FA remains soluble across pH values due to its high oxygen functional group content, whereas HA becomes insoluble in acidic environments. Additionally, HA has a higher nitrogen concentration than FA (Canellas et al., 2015; Islam et al., 2020). Both act as natural colloids and chelating agents, being essential in the complexation, mobilization, and transport of metals, nutrients, and pollutants in edaphic and aquatic systems (Gaffney et al., 1996; Mora et al., 2018; Guo et al., 2019; Islam et al., 2020). The application of these biostimulants to the soil improves soil structure and water- and nutrient-retention capacity, increases cation exchange capacity, and stimulates root growth (Canellas et al., 2001; García et al., 2019; Guo et al., 2019; Machado et al., 2020).

Several studies have shown that these biostimulants promote nutrient uptake and enhance plants’ physiological preparation to mitigate stress. In some cases, the application of FA and HA increased productivity and fruit weight at harvest (Popescu and Popescu, 2018; Zanin et al., 2018; Nunes et al., 2019; Irani et al., 2021). The impact of HA in blueberries varies depending on the cultivar, production system and soil characteristics. Previous studies have suggested that applying HA can promote root growth in certain cultivars (Bryla and Strik, 2015). However, in hydroponic systems, applications via drainage altered the chemical and biological characteristics of the substrate without consistently affecting root growth, productivity, or the quality of SHB blueberries (Nunez et al., 2023). Recent studies have shown that potassium fulvic acid (PFA) effectively conditions saline soils, enhancing plant height, root activity, and chlorophyll synthesis. It also improved soil quality by enhancing organic matter, nitrogen, and potassium levels, while increasing enzyme activity and beneficial microorganisms, and reducing soil electrical conductivity (Wu et al., 2025).

#### 3.1.2 Protein hydrolysates and other N-containing compounds

Protein hydrolysates (PHs) are an important group of non-microbial plant biostimulants, well-known for their ability to promote germination, initial growth, vegetative development, fruiting, fruit quality, and crop productivity, especially under environmental stress (Colla et al., 2017). These compounds result from the chemical, thermal, or enzymatic hydrolysis of proteins of plant and animal origin, generating complex mixtures of free amino acids, oligopeptides, and polypeptides with high bioactivity (Colla et al., 2017; Moreno-Hernández et al., 2020; Malécange et al., 2023).

The composition of PHs is highly variable and depends mainly on the raw material used and the hydrolysis process employed (Du Jardin et al., 2020). Chemical analyses indicate that the protein/peptide content can vary from 1 to 85% (m/m), while free amino acids represent, on average, 2 to 18% (m/m). The main amino acids present are alanine, arginine, glutamate, glutamine, glycine, leucine, proline, and valine (Calvo et al., 2014; Du Jardin et al., 2020). Among them, glutamate is one of the most prevalent, occurring both in the free form and as part of peptide chains (Albarracín et al., 2016). Accordingly, soil application of glutamic acid has been found to improve plant growth, antioxidant metabolism, and fruit quality in blueberry production (Pérez-León et al., 2023). Indeed, their mechanisms of action are primarily mediated by high levels of free amino acids and bioactive peptides, which function as signalling molecules, nitrogen sources, metal-complexing agents, and antioxidant metabolites (Nardi et al., 2016). Root application of PHs has been shown to increase nutrient use efficiency, promote root system development, and enhance nutrient absorption and assimilation in various crops. Also, foliar and substrate-drainage applications have been shown to elicit hormonal activity (e.g., auxins and gibberellins), which promote germination, vegetative growth, fruit set, and fruit development (Colla et al., 2017).

PHs are generally classified by protein source (animal or plant) and production method (enzymatic or chemical hydrolysis) (Colla et al., 2015). Animal-based materials include by-products from the livestock and agri-food industries, such as epithelial and connective tissues, blood meal, fish processing residues, poultry feathers, and casein. On the other hand, plant-based materials include legume seeds, dehydrated alfalfa forage, and products derived from corn wet-milling and horticultural residues. These biostimulants can be applied to the soil, near the root system, or through foliar application (Colla et al., 2015). Among the amino acid-derived compounds that are present in or associated with PHs, glycine betaine (GB) stands out. This compound plays a recognized role in mitigating various types of stress, and its physiological role is so relevant that it warrants a specific approach in the context of its mechanisms of action (Chen and Murata, 2011; Du Jardin, 2015).

##### 3.1.2.1 Glycine betaine

Glycine betaine (GB; N,N,N-trimethylglycine) is a quaternary ammonium compound that carries both positive and negative charges and is electrically neutral at physiological pH. It is synthesized by the oxidation of choline or the stepwise N-methylation of glycine in a variety of species, including plants (Hanson and Rhodes, 1983; Sakamoto and Murata, 2002; Chen and Murata, 2008), where it acts as a compatible solute that promotes osmoregulation and cellular homeostasis during stress. Significant scientific research has established that GB is a highly effective compound for promoting cellular stress tolerance against numerous abiotic stresses (e.g., drought, salinity, frost, heat, oxidative stress). Its protective mechanisms include the stabilization of cellular membranes and large molecular complexes, as well as photosynthetic enzymes and structures (e.g., Rubisco, Photosystem II (PSII)), along with the indirect reinforcement of plant antioxidant defence mechanisms (Chen and Murata, 2011; Hayes et al., 2020).GB enhances physiological performance in plants through an increase in chlorophyll content, net photosynthetic rate, stomatal conductance, relative water content (RWC), water-use-efficiency (WUE), and through a reduction in electrolyte leakage and oxidative damage by the modulation of antioxidant-related enzymes (Ahmed et al., 2019; Hamani et al., 2020; Hafez et al., 2021; Shemi et al., 2021). This osmolyte is also rapidly absorbed by leaves and translocated throughout the plant, supporting its use as a foliar-applied biostimulant (Chen and Murata, 2011). Beyond stress mitigation, GB has been shown to regulate growth and reproductive development. Plants treated with GB alter their carbohydrate metabolism and source-sink relationships, enabling them to produce larger fruits in both stressed and non-stressed conditions. These results were correlated with improved assimilate transport and upregulation of genes involved in photosynthesis, sugar metabolism, hormone signalling, cell division, and cell elongation. Some of the affected hormone signalling pathways include those related to auxins, cytokinins, gibberellins, brassinosteroids, and sucrose transport (Zhang et al., 2019).

In addition to promoting the growth of several horticultural crops, exogenously applied GB significantly increased fruit yield and quality, as well as the accumulation of bioactive compounds, including phenolic compounds, flavonoids, and anthocyanins (Mäkelä et al., 1998; Mickelbart et al., 2006; Jalil and Sabir, 2017; Correia et al., 2019; M. Li et al., 2019a; Gonçalves et al., 2020; Graziani et al., 2022; Serapicos et al., 2022; Monteiro et al., 2023). In blueberries, evidence supporting the use of GB as a biostimulant has increased in recent years. When applied externally, GB activates proline metabolism and maintains the levels of TA, ascorbic acid, and sugars. It also delays cell wall and carbohydrate degradation by coordinating the regulation of enzymes and genes involved in the stress response and fruit softening (Zhang et al., 2025). Further field research confirmed that foliar GB applications increase the yield and size of NHB blueberry fruits, as well as their firmness, TSS, and sweetness (Lopes et al., 2024). More recently, Lopes et al. (2026) found that GB significantly affects the TPC of ‘Duke’ and ‘Draper’ blueberry cultivars, as well as their anthocyanin content and AC. However, they also observed year- and/or cultivar-dependent effects on the vitamin C content of these fruits, demonstrating that GB can influence the complex metabolic pathways involved in antioxidant production in blueberries.

#### 3.1.3 Seaweed extracts

Seaweeds have been used as biostimulants to improve agricultural soils since antiquity and are increasingly important in the agricultural industry (El Boukhari et al., 2020; Stirk et al., 2020). Algae-based products are among the most cost-effective biostimulants available on the market (Rouphael and Colla, 2018), being biodegradable, safe for human health and the environment, economically viable, and widely used to promote crop growth and development (Chrysargyris et al., 2018; Del Buono, 2021). At low concentrations, these extracts enhance plant productivity without harmful side effects and without interfering with land use for food production (Sharma et al., 2014; Deolu‐Ajayi et al., 2022).

There are more than 9,000 species of macroalgae, which are divided into three categories based on pigmentation: brown algae (*Phaeophyta*), red algae (*Rhodophyta*), and green algae (*Chlorophyta*) (Khan et al., 2009). Brown algae, which include approximately 2,000 species, are most commonly used in the production of biostimulants, with *Ascophyllum nodosum*, *Ecklonia maxima*, and *Sargassum* spp. standing out (Blunden and Gordon, 1986; Khan et al., 2009; Tuhy et al., 2013). Algal polymers are structurally complex and chemically diverse, and their composition varies depending on the species, extraction method, location, temperature, and harvest season, factors that influence their effectiveness (Guinan et al., 2013; Shukla et al., 2019; Valverde et al., 2022). These extracts can be obtained by methods such as grinding, acid or alkaline hydrolysis, low-temperature/high-pressure cell disruption, or crushing frozen algae (Stirk et al., 2020).

Seaweeds contain various nutrients, vitamins, amino acids, peptides, phytohormones (e.g., auxins, cytokinins, ABA, gibberellins, ethylene, and brassinosteroids), polysaccharides, fatty acids, omega-3, carotenoids, and phenolic compounds (Khan et al., 2009; Kadam et al., 2015; Stirk et al., 2020). Among phenolic compounds, green and red algae have high amounts of phenolic acids and terpenoids, flavonoids, mycophorin-type amino acids, and bromophenols, while brown algae predominantly contain phlorotannins (Cotas et al., 2020). Phlorotannins have an elicitor effect by stimulating the biochemical pathways involved in the synthesis of phenolic compounds, such as the shikimate/phenylpropanoid pathway, and by inhibiting IAA-oxidase activity (Stirk et al., 2020). In addition, seaweed includes photosynthetic pigments with bioactive properties, such as xanthophylls, carotenoids, chlorophylls (Aryee et al., 2018) and betaine compounds (Blunden et al., 2010), which reduce osmotic stress and leaf chlorophyll degradation as compatible solutes (Khan et al., 2009). The carbohydrates found in algae include laminarin, mannitol, fucoidan, alginate, agar, carrageenans, ulvan, cellulose, and hemicellulose, which can act as molecular priming agents in extracts and induce different physiological responses in plants (Stiger-Pouvreau et al., 2016; Goñi et al., 2020; Kerchev et al., 2020; Rioux and Turgeon, 2015; Shukla et al., 2019), while also performing structural or cellular reserve functions (Stiger-Pouvreau et al., 2016).

Algae-based biostimulants improve soil health and promote plant growth by enhancing soil structure and moisture retention, and by stimulating beneficial microbiota (Khan et al., 2009). They uniquely facilitate the uptake and availability of existing and applied nutrients (El Boukhari et al., 2020), a process that can be enhanced by chelating agents, such as mannitol (Hosseini et al., 2024). In addition to the beneficial effects on soil structure, the use of these biostimulants is associated with root system development, including the formation of root hairs and secondary roots, as well as increased plant growth (Mukherjee and Patel, 2020), which can be explained by the presence of auxins (Jeannin et al., 1991; Crouch et al., 1992), although the low hormone concentrations present in commercial products suggest that the physiological effects result mainly from the modulation of endogenous hormone homeostasis and molecular network interactions, and the stimulation of biosynthetic processes (De Saeger et al., 2020; Stirk et al., 2020; Baltazar et al., 2021). In addition to improving physiological parameters and crop yields, seaweed extracts promote crop tolerance to various abiotic stresses (e.g., heat, frost, drought, salinity), promoting plant growth under these conditions (Guinan et al., 2013; Shukla et al., 2019; De Saeger et al., 2020; Mukherjee and Patel, 2020; Deolu‐Ajayi et al., 2022; Han et al., 2024; Lenart et al., 2024). Specifically, the application of seaweed extracts to blueberries has been shown to mitigate the negative effects of water stress, enhancing drought resistance and activating antioxidant mechanisms. This was evidenced after the application of algae-based biostimulants to the ‘Brigitta Blue’ cultivar, with a significant increase in catalase (CAT) activity, with average increases of around 20% (Lenart et al., 2024). Under normal growing conditions, foliar treatments with *Ascophyllum nodosum* extracts promoted shoot elongation in ‘Biloxi’ blueberry plants (García-Vázquez et al., 2023), while application via fertigation stimulated vegetative growth in the ‘Draper’ cultivar (Bryla and Vargas, 2023). In addition to growth effects, several studies report consistent improvements in fruit yield and quality after the application of seaweed-based biostimulants, including increases in fruit weight and firmness, TSS and sugars, TPC, anthocyanins, and AC in different blueberry cultivars (Loyola and Muñoz, 2009; Koort et al., 2020; Lenart et al., 2022a, 2022b; Paraschiv et al., 2023). Recent studies indicate additional benefits when algae-based biostimulants are combined with other compounds. The joint application of *Ascophyllum nodosum* with other biostimulants, such as algae biostimulant (NPK + TE), chitosan, and mycorrhizae, resulted in significant improvements in the mineral profile, vitamin C content, dry matter, and TSS of ‘Duke’ blueberry fruits, suggesting synergistic effects in optimizing nutritional and functional quality (Krasniqi et al., 2025). Consistently, Lopes et al. (2024) demonstrated that foliar applications of *Ecklonia maxima* (EM) substantially increased the yield, weight, size, and firmness of fruits from cultivars such as ‘Duke’ and ‘Draper’, with responses dependent on the dose applied. A recent study indicates that higher doses (4 L ha^-1^) of EM were more effective in increasing the concentration of anthocyanins, phenolic compounds, and antioxidant activity in blueberries (Lopes et al., 2026). Additionally, these authors demonstrated that the combined application of EM and GB was particularly promising for improving the AC of the fruit. These effects reflect integrated regulation of primary and secondary metabolism, with a positive impact on both the fruit’s commercial quality and its nutraceutical value.

#### 3.1.4 Chitosan and biopolymers

The application of naturally occurring biopolymers, such as cellulose, collagen, alginate, chitin, and chitosan, has been reported to have biostimulant potential (Palacio-Márquez et al., 2022). Among these, chitosan has received particular attention due to its high biological and agronomic versatility. Chitosan is a copolymer consisting of D-glucosamine and N-acetyl-D-glucosamine units, obtained by partial deacetylation of chitin, a polysaccharide found in the cell walls of crustaceans, fungi, insects, some microorganisms, algae, and invertebrates (Malerba and Cerana, 2016; Pellis et al., 2022; Stasińska-Jakubas and Hawrylak-Nowak, 2022). The degree of deacetylation and molecular weight are factors that strongly influence the physicochemical and biological properties and determine its solubility, reactivity, and biological efficacy (Pellis et al., 2022; Riseh et al., 2022; Stasińska-Jakubas and Hawrylak-Nowak, 2022; Román-Doval et al., 2023). Currently, size-controlled chitosan polymers and oligomers are available on the market for use in the food, cosmetic, medical, and agricultural sectors (Du Jardin, 2015). In agriculture, chitosan was initially used for protection against fungal pathogens. However, its use has been expanded to induce tolerance in plants to various abiotic stresses, as well as to regulate primary and secondary metabolism and fruit quality (Du Jardin, 2015). The application of chitosan has shown several benefits, such as promoting plant growth and crop yield, improving pulp firmness, and stimulating root system development (Soppelsa et al., 2019). The physiological effects of chitosan include modulation of photosynthetic activity, improved CO_2_ assimilation efficiency, increased accumulation of bioactive compounds and AC, with responses that generally vary according to genotype, applied dose, and characteristics of the polymer used (Griñán et al., 2019; Salachna et al., 2025; Reyes-Pérez et al., 2025). In addition, foliar application of this polymer stimulates the absorption and redistribution of micronutrients, improves mineral nutrition, and enhances fruit biofortification (Soppelsa et al., 2019; Sathiyabama and Manikandan, 2021; El Amerany et al., 2022).

From a phytosanitary perspective, chitosan acts through direct antimicrobial activity against pathogens, including damage to the plasma membrane, electrostatic interactions with DNA and RNA, metal chelation, and deposition on microbial surfaces. Another mechanism involves inducing plant defence responses, mediated by signalling cascades, transcription factor activation, and gene expression, upon recognition of chitin and chitosan by cell membrane receptors (Riseh et al., 2022). Simultaneously, this biopolymer is associated with the activation of enzymatic systems that detoxify ROS, which involve signalling molecules such as hydrogen peroxide and nitric oxide. In addition, it can interact with chromatin and directly influence gene expression (Malerba and Cerana, 2016).

In blueberries, chitosan has been shown to have a very significant effect on postharvest quality and preservation. For example, the application of chitosan increased the shelf life of blueberries by up to 25 days after harvest, thereby limiting deterioration and maintaining commercial quality (Lepaja et al., 2024). Additionally, preharvest application trials across different blueberry cultivars have shown that chitosan acts as a defence elicitor, promoting the accumulation of phenolic compounds, anthocyanins, and the AC of the fruit, with consistent effects across years of production (Cola et al., 2024). Investigations with highbush blueberries have also shown that the chitosan’s degree of deacetylation and molecular mass influence the effectiveness of the treatment. High-molecular-weight chitosan was most effective in improving the fruit’s physical and chemical parameters (e.g., firmness, intense blue coloration, ascorbic acid content, and phenolics, particularly anthocyanins) and in reducing mycotoxin contamination (Figiel-Kroczyńska et al., 2022).

#### 3.1.5 Silicon as an inorganic compound and beneficial element

The definition of beneficial elements must consider the specific conditions under which they can exert a positive effect on plant development and stress response, rather than solely their chemical properties (Du Jardin, 2015). In this context, Si has been recognized as beneficial to plants (Ma et al., 2001). After oxygen, it is the most abundant element in the Earth’s crust and is absorbed by plants in the form of monomeric or monosilicic acid (H_4_SiO_4_) from the soil solution, at concentrations ranging from 0.1 to 0.6 mmol L^-1^. In plants, Si content varies considerably from 0.1% to 10% of the DW (Epstein, 1994; Ma and Yamaji, 2006; Liang et al., 2007), which can equal or exceed the three primary macronutrient levels. Due to the ascending flow of the transpiration system, its concentration is usually higher in transpiration organs, such as leaves (Meena et al., 2014; Mandlik et al., 2020).

Following uptake and translocation, Si is deposited in plant tissues as SiO_2_ phytoliths, which may form on a carbohydrate matrix within the cell walls or be deposited inside the cell lumen. Therefore, phytoliths can be found in the epidermis of leaves, stems, and inflorescence bracts as well as in the endodermis of roots, with a minor quantity present in seeds (Hodson, 2019). According to Savvas and Ntatsi (2015), these substances can promote plant rigidity and control the movement of water and nutrients. Additionally, the deposition of a Si layer beneath the cuticle can mitigate drought stress by reducing leaf transpiration (Ma et al., 2001). The Si-containing cellulose membrane in the epidermis also helps prevent excessive water loss by reducing stomatal pore size (Meena et al., 2014).

By complexing or co-precipitating toxic metals in plant tissues and soil, Si immobilizes them and controls nutritional deficiencies and toxicity in plants (Savvas and Ntatsi, 2015; Pavlovic et al., 2021). The reduction in metal-binding sites caused by Si deposition in roots decreases the absorption and translocation of toxic metals and salts (Meena et al., 2014). Si can also regulate the phytohormones of plants subjected to stress conditions, such as ABA, jasmonic acid (JA), and salicylic acid (SA), affecting gene signalling and expression and enhancing crop antioxidant defence mechanisms, increasing the activity of key ROS-scavenging enzymes such as superoxide dismutase (SOD), CAT, glutathione reductase (GR), glutathione peroxidase (GPX), ascorbate peroxidase (APX), and dehydroascorbate reductase (DHAR) (Meena et al., 2014; Savvas and Ntatsi, 2015; Khan et al., 2023). Furthermore, Si regulates secondary metabolic processes, leading to up-regulation of genes involved in the phenylpropanoid pathway, thereby increasing the production and accumulation of phenolic compounds with antioxidant, antimicrobial, and structural protection (Ahanger et al., 2020).

This element is essential to plants for physiological and metabolic activities as it helps plants withstand biotic, activating triggering plant defence responses to herbivores and fungal infections, and abiotic stresses (e.g., soil salinity and heat), increases crop productivity by promoting photosynthesis, improves the number of commercial fruits, fruit weight, size, and firmness and helps plants absorb water and uptake and translocate nutrients (Liang et al., 2007; Harizanova et al., 2014; Liang et al., 2015; Muneer et al., 2017; Ouellette et al., 2017; Gomes et al., 2020; Frew et al., 2018; Costan et al., 2020; González-Terán et al., 2020; Santos et al., 2020; Karagiannis et al., 2021). Additionally, Si stimulates lateral root elongation in plants, potentially by increasing cell wall extensibility (Hattori et al., 2003; Isa et al., 2010), and promotes growth and crop nutrient balance, with the beneficial effects being more visible in stressed plants (Cooke and Leishman, 2016; Etesami and Jeong, 2018).

In blueberries, applying Si to ‘Ventura’ bushes via a nutrient solution increased their uptake of water and nutrients (K^+^ and NO_3_^-^), as well as promoting stem, leaf, and fruit growth, and increasing TSS, while both foliar spray and nutrient solution applications increased leaf area, fruit yield, and firmness (Ferrón-Carrillo et al., 2022; Zydlik et al., 2022). The foliar spray of Si also increased the concentration of nitrogen and copper in the leaves, as well as the weight and size of ‘Bluecrop’ fruits (Zydlik et al., 2022). Adding this element to the growing medium increased plant FW and reduced proline and malondialdehyde levels in ‘Liberty’ cultivar (Figiel-Kroczyńska et al., 2022). Furthermore, fertigation with 1.2 mmol L^-1^ boosted plant height by 8% while increasing dry and fresh shoot biomass by 21% and 25%, respectively, and increased vegetative development in ‘Ventura’ blueberries by 8-25% (Gallegos-Cedillo et al., 2018). The application of this compound has recently been shown to improve fruit quality and photosynthesis in several blueberry cultivars by increasing vegetative growth, yield, leaf area, chlorophyll fluorescence, postharvest weight and diameter, TA, phenolics, and anthocyanins, and decreasing peroxidase activity (Silva et al., 2023).

#### 3.1.6 Beneficial fungi and bacteria

The application of beneficial microorganisms has consistently exhibited positive effects on plant physiology and growth, mineral nutrition, and resistance to biotic and abiotic stress in blueberries. However, it is essential to use bioinoculants with high biosafety, as inappropriate microorganisms can pose risks to public health and the environment (Nunes et al., 2022).

Plant growth-promoting bacteria (PGPB) are characterized by their wide availability, their capacity to adapt to diverse environmental conditions, and their potential as biocontrol agents (Hafez et al., 2021; Wang and Yang, 2024). In blueberries, root irrigation with PGPB of the genera *Pseudomonas* and *Buttiauxella* led to considerable increases in the number of branches and leaves, chlorophyll content, and plant height compared to the control (Wang and Yang, 2024). These effects were associated with phosphorus solubilization, auxin production, and increased soil fertility, thereby promoting a positive change in the rhizosphere microenvironment and microbial community structure. In addition, bacterial endophytes and PGPB are known to enhance vegetative growth, suppress grey mould, and improve fruit yield and quality traits in various blueberry cultivars. Beneficial effects on plant performance included increased biomass, chlorophyll content, fruit firmness, TPC, anthocyanins, organic acids, and AC (Contreras-Pérez et al., 2019; Karaklajić-Stajić et al., 2023; Flores-Félix et al., 2024).

Blueberries also benefit from associations with various beneficial fungi, including *Trichoderma* spp., ericoid mycorrhizal fungi (ErM), yeast species, fungal endophytes, and non-virulent strains of specific pathogens (Ghorbanpour et al., 2018). The majority of important ErM groups belong to the Ascomycota, including the *Hyaloscypha hepaticicola* complex. This complex includes *H. hepaticicola*, *Meliniomyces variabilis*, *Oidiodendron maius*, and species of the genus *Leohumicola* (Fehrer et al., 2019; Vohník, 2020). Inoculation with ErM fungi can improve root structure, plant growth, and nutrient uptake in blueberries. For example, *Oidiodendron maius* stimulated root growth and adventitious root formation (Wei et al., 2022), as well as causing distinct changes in the fibrous and pioneer roots of *Vaccinium virgatum* ‘Tifblue’, depending on the strain used (Baba et al., 2021). The EF1409 strain favoured greater pioneer root growth, whereas EF1453 promoted lateral root formation, thereby promoting efficient mycorrhizal colonization and symbiotic function (Baba et al., 2021). Besides promoting root development and biomass accumulation, soil inoculation with this fungal species reduced aluminium uptake and enhanced cation exchange capacity under controlled growth conditions (Yang and Goulart, 2000; Baba et al., 2021). In conventional mycorrhizal interactions, the host plant donates photoassimilates to the fungus in exchange for increased access to water and mineral nutrients, promoting mutual benefit (Wang et al., 2017b; Tedersoo and Bahram, 2019; Genre et al., 2020; Shi et al., 2023b). In their natural state, plants are colonized by complex communities of microorganisms whose interactions influence both host health and the functioning of microbial consortia in the rhizosphere (Hu et al., 2016). In blueberries, microbial consortia used alone or in conjunction with humic substances resulted in an increase in both aerial and root biomass, as well as more efficient nutrient uptake (Schoebitz et al., 2016, 2019). Arbuscular mycorrhizal fungi (AMF) are also important in agriculture, as their extensive network of external hyphae can greatly increase the surface area and volume of soil explored, thereby increasing nutrient uptake (Giovannetti et al., 2001). These symbioses can modulate the primary and secondary metabolism of plants, stimulating the production of phytochemicals in roots and aerial organs (Sbrana et al., 2014), thereby driving the growing interest in using AMF as biostimulants in sustainable horticultural systems (Rouphael et al., 2015). Additionally, the manipulation of endophytic fungi has proven to be a viable strategy to improve blueberry resilience. The application of Antarctic fungal endophytes simultaneously improved tolerance to cold and drought stress, which can be attributed to increased photochemical efficiency, reduced oxidative stress, upregulation of stress tolerance-associated gene expression, and increased plant survival (Acuña-Rodríguez et al., 2022).

### 3.2 Effects of calcium application on blueberry fruit quality

Alongside biostimulants, preharvest Ca application has been widely explored as a strategy to improve blueberry yield and fruit quality (**Table 1**). Compared to other temperate fruit crops, blueberries are considered calcifuge because they thrive in acidic, low-calcium soils (Retamales and Hancock, 2018). However, Ca is an important nutrient for the stability of cell walls and the binding of cell wall polysaccharides (e.g., pectin) and affects cell wall extensibility and membrane structure, which allows intracellular communication and controls the vacuolar osmotic balance (Hocking et al., 2016). Blueberry firmness is positively influenced by cell wall polysaccharide composition, including de-esterified pectins (homogalacturonans) and xyloglucans, as well as cell wall-associated Ca^2+^, which stabilizes pectin structure and modulates the activity of cell wall-degrading enzymes such as polygalacturonase (PG) and pectin methylesterase (PME) (Martins et al., 2018; Olmedo et al., 2021). Ca also acts as a secondary messenger in plants, regulating specific hormone signal transduction pathways associated with growth and stress responses (Hocking et al., 2016). Once absorbed, Ca^2+^ is rapidly compartmentalized into vacuoles, endoplasmic reticulum, organelles, or the apoplast, allowing fine control of cell signalling (Conn and Gilliham, 2010). In addition, auxins facilitate the movement of these ions by altering cell transport mechanisms, promoting cell wall acidification and cell expansion. However, excessive Ca concentrations can inhibit growth by competing with protons for binding sites (Hocking et al., 2016). Beyond its structural role, Ca also contributes to metabolic regulation, including the stimulation of TPC synthesis in fruits (Vighi et al., 2019). Moreover, early foliar applications of Ca increase the capacity to neutralize free radicals, thereby reducing lipid peroxidation and oxidative stress in cell membranes (Lobos et al., 2021a).

**Table 1:** Studies reporting the effects of preharvest biostimulants and selected agronomic inputs on vegetative growth, yield components, physiological traits, and fruit quality attributes of blueberry.

Biostimulant/input	Application method	Experimental setup	Cultivar	Main recorded effects	References
Humic acid (HA)	Soil application/foliar spray	Field	Legacy	Increased total phenolic content (TPC) by 65% and antioxidant capacity (AC)	Schoebitz et al. (2019)
Humic and fulvic acids (HA + FA)	Foliar spray	Field	Draper; Bluecrop	Promoted root development and overall plant growth	Bryla and Vargas (2023)
Potassium fulvic acid (PFA)	Soil drench	Greenhouse/pots	Reka	Enhanced plant height, root activity, and chlorophyll synthesis under saline stress; improved soil organic matter, N and K availability, enzymatic activity, and beneficial microbial populations; reduced soil electrical conductivity	Wu et al. (2025)
Microbial consortium (± HA)	Soil application/foliar spray	Field	Legacy	Increased shoot dry weight, plant height, root biomass, fruit yield, nutrient accumulation (N, K), TPC, and AC	Schoebitz et al. (2016, 2019)
Plant growth-promoting bacteria (PGPB)	Root inoculation	Field	Duke; Legacy	Increased fruit yield, weight, size, and firmness; reduced titratable acidity (TA); increased maturity index (MI), lightness (L*), TPC, and AC	Flores-Félix et al. (2024)
Ericoid mycorrhizal and endophytic fungi	Root inoculation/soil application	Field/greenhouse/pots	Multiple cultivars	Consistently enhanced root development, plant biomass, nutrient uptake (N, P, K, Mn, Ca, Zn), flowering, and fruit weight and yield, reflecting functional specialization of ericoid mycorrhizal associations in *Vaccinium* spp.	Scagel (2005); Bizabani et al. (2016); Brody et al. (2019); Guo et al. (2021); Li et al. (2021b); Ważny et al. (2022)
Antarctic fungal endophytes	Root inoculation	Outdoor pots	Brigitta	Improved photochemical efficiency; reduced lipid peroxidation; enhanced tolerance to cold and drought stress; increased fruit weight and size	Acuña-Rodríguez et al. (2022)
Mycorrhizal fungi	Root application	Field/pots	Duke	Increased flower number and fruit yield	Lepaja et al. (2024)
Fungal species (*Oidiodendron maius*)	Root inoculation	Controlled growth room/solution culture	Elliott; Tifblue	Increased root and leaf dry weight; improved root development; reduced Al uptake; increased cation exchange capacity	Yang and Goulart (2000); Baba et al. (2021)
Biovermix^®^ (*Azotobacter* sp., *Bacillus* sp., *Pseudomonas* sp., *Trichoderma* sp.)	Soil drench	Field	Aurora	Increased fruit TPC, anthocyanins, and organic acids	Karaklajić-Stajić et al. (2023)
Chitosan	Foliar spray	Field	Sunrise	Increased fruit N-NO_3_^-^ and N-NO_2_^-^, weight, firmness, TSS; reduced TA; increased L*, TPC, anthocyanins, vitamin C, and AC	Figiel-Kroczyńska et al. (2022)
Chitosan	Foliar spray	Pots	Cosmopolitan; Hortblue Poppins; Legacy	Increased accumulation of phenolic compounds, anthocyanins, and AC	Cola et al. (2024)
Stymjod^®^ (macro- and micronutrients + humic and amino acids)	Foliar spray	Field	Bluecrop	Increased leaf N, Zn, Cu, and B; enhanced chlorophyll and carotenoids; increased leaf area; increased fruit Zn, Cu, and B; increased fruit yield, weight, size, and firmness	Zydlik et al. (2022)
Plant growth-promoting rhizobacteria (PGPR)	Root irrigation	Pots (soil from blueberry field)	–	Increased plant height, number of branches and leaves, root length, and chlorophyll content	Wang and Yang (2024)
Bacterial endophytes (PGPB)	Soil inoculation	Greenhouse	Biloxi	Exhibited antifungal activity against grey mould; increased shoot length and biomass; enhanced root biomass and chlorophyll content	Contreras-Pérez et al. (2019)
Chitosan + Seaweed extract (*Ascophyllum nodosum*)	Foliar spray	Field/pots	Duke	Increased canopy volume and number of flowers and fruits	Lepaja et al. (2024)
Amino acids (glutamic acid)	Soil drench	Field	Biloxi	Increased bud number; enhanced chlorophyll, TPC, flavonoids, antioxidant enzymes (GPX, CAT); increased fruit weight and size, TSS, anthocyanins, vitamin C; reduced PAL activity	Pérez-León et al. (2023)
Plant growth regulators (auxins, cytokinins, gibberellins, ABA, jasmonates, brassinosteroids)	Foliar spray/dipping/soil application	Field/greenhouse	Multiple cultivars	Modulated vegetative growth, flowering, fruit set, yield, and fruit quality in a hormone- and phenology-dependent manner, influencing shoot architecture, leaf area, carbohydrate accumulation, firmness, TSS, and secondary metabolism	Buran et al. (2012); Zhang et al. (2017); Oh et al. (2018); Milić et al. (2018); Cappai et al. (2020); Pérez-León et al. (2023)
Dikegulac (2,3:4,6-di-O-isopropylidene-α-Lxylo-2-hexulofuranosonic acid) + Asahi SL (sodium *ortho*- and para-nitrophenolate, sodium 5-nitroguaiacolate)	Foliar spray	Pots (foil tunnel)	Bluecrop; Brigitta Blue; Darrow	Increased shoot number; altered shoot length distribution; increased chlorophyll index (SPAD) and photosynthetic efficiency (Fv/Fm, Fv/Fo)	Litwińczuk (2023)
Melatonin	Foliar spray	Field	Brigitta	Increased soluble sugar content	Zhang et al. (2017)
Silicon	Woody plant medium	*In vitro*	Liberty	Increased plant fresh weight; reduced proline and lipid peroxidation (MDA)	Figiel-Kroczyńska et al. (2022)
Silicon	Nutrient solution	Greenhouse	Ventura	Increased nutrient and water uptake; enhanced vegetative growth; increased fruit yield, size, TSS, and firmness	Ferrón-Carrillo et al. (2022)
Silicon	Foliar spray	Field	Brightwell; Climax; Beckblue	Enhanced chlorophyll fluorescence parameters and PSII efficiency; increased leaf area, fruit yield, fruit TPC, and anthocyanins; reduced POD activity	Silva et al. (2023)
Silicon	Foliar spray	Field	Bluecrop	Increased leaf N and Cu; increased fruit yield, size, and firmness	Zydlik et al. (2022)
Silicon	Nutrient solution	Greenhouse	Ventura	Increased shoot biomass	Gallegos-Cedillo et al. (2018)
Titanium organic complex	Foliar spray	Field	Bluecrop	Increased pollen germination, seed number, fruit set, fruit size, and weight	Bieniasz and Konieczny (2022)
Seaweed extracts (*Ascophyllum nodosum*)	Foliar spray	Greenhouse/pots	Biloxi; Draper	Stimulated vegetative growth	Bryla and Vargas et al. (2023); García-Vázquez et al. (2023)
Seaweed extracts (*Ascophyllum nodosum*)	Foliar spray	Field	O’Neal’	Increased SST and fruit size	Loyola and Muñoz (2009)
Seaweed extract (*Ecklonia maxima*)	Foliar spray	Field	Duke; Draper	Increased fruit yield, size, weight, and firmness; enhanced anthocyanin and phenolic contents and AC; synergistic effects reported when combined with glycine betaine (GB)	Lopes et al. (2024); Lopes et al. (2026)
Algae biostimulants (NPK + TE)	Foliar spray	Outdoor pots	Duke	Increased canopy volume	Lepaja et al. (2024)
*Ascophyllum nodosum* + algae biostimulant (NPK + TE) + chitosan + mycorrhizae	Foliar spray/root application	Field/pots	Duke	Improved mineral profile, vitamin C content, dry matter, and TSS of fruits	Krasniqi et al. (2025)
Seaweed extracts	Foliar spray	Field	Brigitta Blue; Bluecrop	Increased fruit weight, firmness, delfinidin-3-galactoside, TPC, AC, and catalase; decreased peroxidase concentrations	Lenart et al. (2022b, 2022a, 2024)
Seaweed extracts	Soil application (granular)	Abandoned peat field	Northblue	Increased fruit yield, tritatable acidity, and anthocyanins; decreased MI	Koort et al. (2020)
Seaweed extracts	Foliar spray	Field (plants grown on polyethylene-covered drums)	Duke; Delicia; Simultan; Elliott; Safir; Pastel; Vital	Increased fruit yield and weight, TSS, total sugars, firmness, and total acidity	Paraschiv et al. (2023)
Glycine betaine	Foliar spray	Field	Duke; Draper	Increased fruit yield, size, and firmness, as well as TSS and sweetness; increased TPC, anthocyanins, and AC; synergistic effects reported with EM	Lopes et al. (2024); Lopes et al. (2026).
Calcium	Foliar spray	Field	Bluecrop; Duke	Increased leaf N, Ca, Mg, Zn, and Cu concentrations, SPAD values, and leaf area; increased fruit N (including NO_3_^-^), Ca, Zn, and Cu concentrations, fruit yield, firmness, weight, size, TPC, and pH; reduced TA	Ochmian (2012); Zydlik et al. (2022)
Calcium	Soil application	Field	Jersey; O’Neal; Bluecrop	Increased Ca concentration in leaves and fruits; enhanced fruit firmness and Na_2_CO_3_-extractable fractions; reduced water-soluble neutral sugars and uronic acids	Hanson and Berkheimer (2004); Angeletti et al. (2010)
Calcium	Tissue culture	*In vitro*	Legacy; Farthing	Increased leaf number	Cappai et al. (2020)
Calcium	Field	Foliar spray	Draper	Increased fruit yield and weight; reduced yield loss and fruit drop	Gerbrandt et al. (2019)
Calcium	Field	Foliar spray	Liberty	Increased fruit AC, TPC, and firmness	Lobos et al. (2021a); Lobos et al. (2021b)
Calcium	Field	Foliar spray	Sunrise; Brigitta	Increased fruit TPC, firmness, and weight; reduced TA; increased anthocyanins	Ochmian and Kozos (2014)
Calcium	Field	Foliar spray	Alapaha; Bluecrop; Powder Blue	Increased fruit firmness, height and weight	Koron et al. (2009); Smith (2016)
Calcium	Field	Foliar spray/Soil application	Elliott	Preharvest Ca applications improved fruit firmness compared with untreated plants	Manríquez et al. (2011)

AC, antioxidant capacity; CAT, catalase; FA, fulvic acids; GB, glycine betaine; GPX, glutathione peroxidase; HA, humic acid; MI, maturity index; MDA, malondialdehyde; PFA, potassium fulvic acid; PGPR/PGPB, plant growth-promoting rhizobacteria/bacteria; POD, peroxidase; PSII, photosystem II; SPAD, chlorophyll index; TSS, total soluble solids; TA, titratable acidity; TPC, total phenolic content. Reported effects refer to statistically significant responses relative to untreated controls, as reported in the original studies..

Plants predominantly absorb this nutrient from the soil at the beginning of the season, and it is then transported to the aerial tissues via transpiration, with deficiency symptoms more likely in younger tissues (Hirschi, 2004; Lara, 2013). In fruits, Ca accumulation depends on water transported by the xylem via the apoplastic pathway from the roots to the leaves, shoots, and fruits (White, 2003). On the other hand, its mobility in the phloem is limited (Hocking et al., 2016), and a reduction in the number of leaves during sprouting can influence Ca absorption (Doyle et al., 2021). As such, fruits may have low Ca levels due to competition between shoots with high transpiration rates and fruits with low transpiration rates (Saure, 2005). This dynamic is especially apparent in blueberries, where high Ca concentrations are common in leaves and low in fruits (Gerbrandt et al., 2019). In addition, stomatal conductance and Ca concentration in blueberry fruits decrease dramatically with fruit growth and ripening. This decrease is attributed to a reduction in stomatal density, as well as the late development of cuticular waxes that cover the stomata, which further reduces the stomatal conductance of the fruit compared to the leaves (Stückrath et al., 2008; Yang et al., 2020). Blueberry cultivars with fewer seeds or those subjected to adverse environmental conditions during pollination exhibit lower Ca levels in the fruit and its epidermis, with Ca concentration generally higher in the seeds than in the pulp (Yang et al., 2020). In general, blueberry leaf tissue typically contains between 0.3% and 0.8% Ca, with 60% of Ca localized in the cell wall (Eck, 1988; Glenn et al., 1988), varying significantly with the cultivar (Strik and Vance, 2015).

The effects of applying Ca to fruit crops depend significantly on the formulation, dose, method, and timing of application, as well as the genotype. In general, increased fruit firmness and reduced susceptibility to deterioration can occur after preharvest application of this mineral, although the effects on other quality parameters are not always consistent (Lara, 2013). For instance, the efficiency of exogenous absorption of Ca depends on the permeability of the tissues at the time of application and the concentration and volume of the spray that contacts the fruit (Gerbrandt et al., 2019). Foliar application of Ca, alone or combined with non-ionic surfactants, can increase the concentration of this nutrient in fruits and improve postharvest quality, although excessive doses can induce phytotoxicity (Lara, 2013; Lötze and Turketti, 2015; Arrington and DeVetter, 2017; Gerbrandt et al., 2019). In blueberries, their absorption by the fruit occurs mainly during the early stages of development, particularly during the initial green stage. As ripening progresses, absorption decreases as a result of the development of cuticular waxes (Gerbrandt et al., 2019; Yang et al., 2020). For this reason, foliar fertilization with Ca can be especially recommended during the first weeks after flowering. Particularly, the application of CaCl_2_ between fruit set and the early green stage significantly increased the firmness of ‘Liberty’ blueberries (Lobos et al., 2021b), while repeated spraying with high concentrations of Ca after flowering reduced fruit drop and increased the Ca concentration in fruits of the ‘Draper’ cultivar (Gerbrandt et al., 2019). Also, applications of CaCl_2_ and Ca_3_(PO_3_)_2_ significantly reduced the percentage of fruit drop and estimated production losses, with CaCl_2_ also promoting a slight increase in average fruit weight (Gerbrandt et al., 2019). Ca-based foliar fertilizers have also been associated with increased fruit size and reduced acidity in ‘Brigitta’ blueberries (Ochmian and Kozos, 2014), as well as increased fruit weight and pH and decreased TA in cv. ‘Duke’ (Ochmian, 2012; Ochmian and Kozos, 2014). In addition, foliar applications increased the height of blueberry plants (Koron et al., 2009; Ochmian, 2012) and improved the firmness and resistance to rupture of the fruit epidermis (Angeletti et al., 2010; Manríquez et al., 2011; Smith, 2016; Ochmian, 2012; Ochmian and Kozos, 2014; Lobos et al., 2021b). On the other hand, foliar or soil application of Ca does not always translate into qualitative increases related to firmness, size, and weight, or even increased Ca concentration in different blueberry cultivars (Arrington and DeVetter, 2017; Hirzel, 2023). In addition to physical effects, Ca applications have been associated with increased concentrations of phenolic compounds and anthocyanins in blueberry fruits (Ochmian and Kozos, 2014) and negatively associated with PG activity (Lobos et al., 2021b). The concentration of Ca in leaves can also be increased by both soil and foliar applications, resulting in higher SPAD values and improved overall nutritional status of the plant (Hanson and Berkheimer, 2004; Ochmian, 2012). Recent studies have also shown that foliar application of Ca-based fertilizers increased leaf area and nutrient status while simultaneously promoting increased yield, firmness, weight, size, and TPC content in ‘Bluecrop’ blueberries (Zydlik et al., 2022).

## 4 Postharvest strategies to preserve blueberry quality

**Figure 1:**
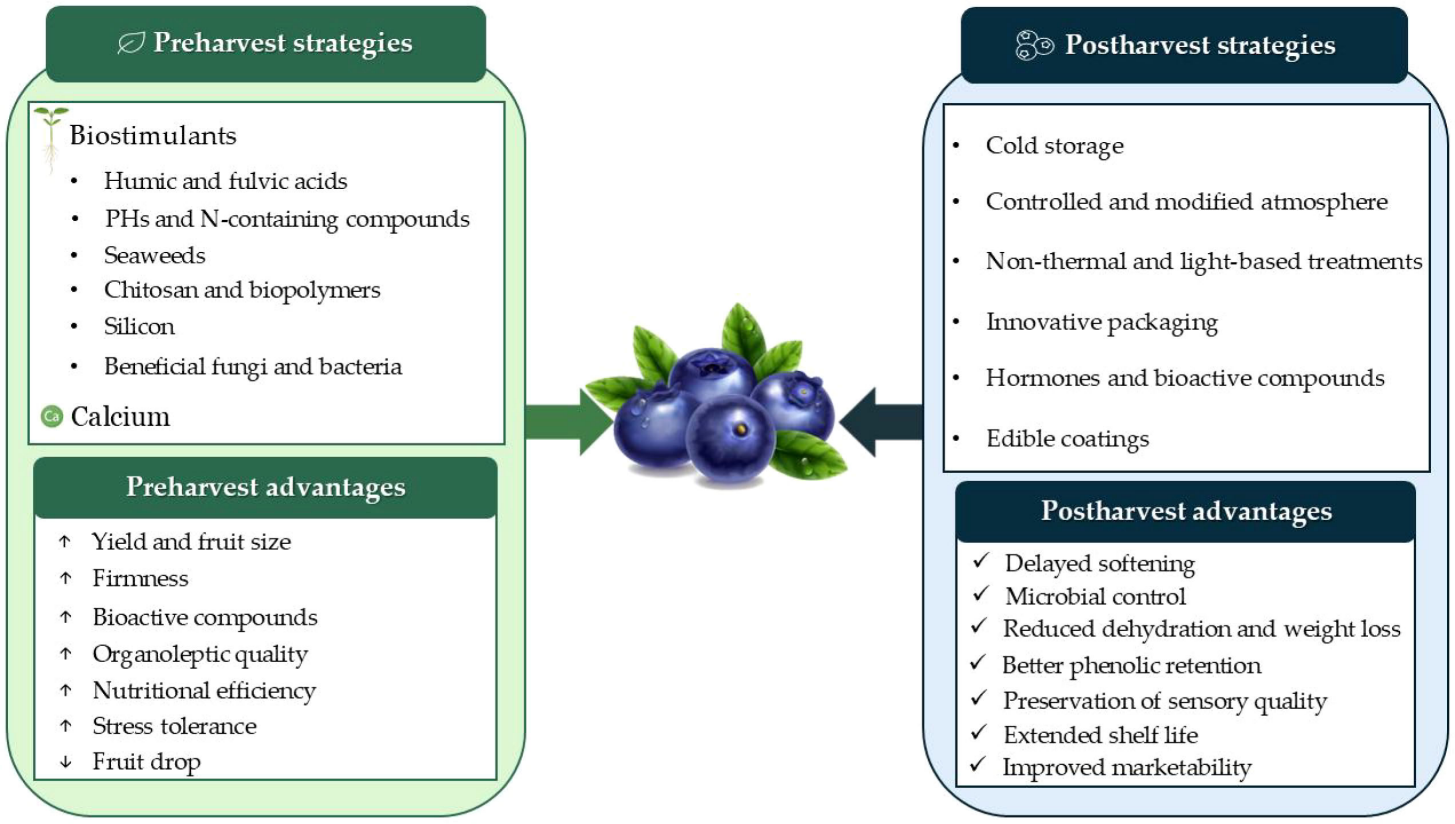
Overview of pre- and postharvest strategies and their main advantages for blueberry quality.

To ensure the quality and shelf life of blueberries during storage, it is important to understand the factors that affect them. Blueberry quality tends to decline during postharvest storage due to physical and biochemical changes, which can be exacerbated by dehydration and fungal infections (Paniagua et al., 2013; Kozos et al., 2014; Vieira et al., 2016). Dehydration affects the fruit texture, as evidenced by a decrease in firmness as dehydration increases (Moggia et al., 2017b; Rivera et al., 2021), with a more pronounced effect in fruits with more extensive peduncular scars (Moggia et al., 2017a) depending on the cultivar and ripeness level (Moggia et al., 2016). As the fruit ripens, a reduction in the cell wall resistance is observed, resulting in decreased hemicellulose content, and enhanced cell wall solubilization (Vicente et al., 2007). Therefore, softening during storage results from the enzymatic degradation of cell wall polysaccharides, particularly pectins, and the hydrolysis of reserves such as starch (Duan et al., 2011; Sun et al., 2022). Pectins, hemicelluloses, and cellulose collectively maintain fruit firmness within the blueberry cell wall. Among pectins, structural variants such as rhamnogalacturonan-I (RG-I) and arabinogalactan proteins are particularly relevant, as firmer cultivars consistently present higher levels of arabinan and type II arabinogalactan side chains that resist enzymatic depolymerisation during ripening and storage (Trandel-Hayse et al., 2023; Sanhueza et al., 2024). Hemicellulosic components (e.g., xyloglucan and heteromannan) further modulate texture by contributing to the mechanical reinforcement of the cell wall network, with their relative abundance varying significantly across cultivars and accounting for part of the observed inter-cultivar variation in firmness (Zhang et al., 2022; Trandel-Hayse et al., 2023). The activity of various pathogens during storage, such as grey mould (*Botrytis cinerea*), anthracnose (*Colletotrichum* spp.), and Alternaria rot (*Alternaria* spp.), causes significant losses due to fruit decay (Bell et al., 2021). As a result, blueberries can develop pitting, which is associated with increased lipid peroxidation, reduced antioxidant enzyme activity, and damage to cell membranes. In consequence, loss of firmness may be associated with ROS accumulation, which leads to oxidative stress and interferes with enzymatic reactions involved in phenolic compound metabolism (Zhou et al., 2014). Comprehending these mechanisms thus entails assessing the suitability of different genotypes for prolonged storage and transport, thereby reducing postharvest losses and increasing the sector’s sustainability and profitability (Giongo et al., 2013). An overview of the pre- and postharvest strategies discussed in this review, along with their main advantages for blueberry quality, is presented in **Figure 1**.

Although blueberries are not considered climacteric fruits, the influence of ethylene on postharvest physiological processes may depend on the genotype. This can result in increases in anthocyanin content and AC, as well as changes in respiratory rates and ethylene production, consistent with the patterns observed in climacteric ripening (Costa et al., 2018; Wang et al., 2022). Genotypic differences in ethylene production affect fruit texture and behaviour during storage, enabling adjustment of pre- and postharvest strategies based on these levels to extend shelf life and preserve quality (Farneti et al., 2020).

This fruit has a complex defence system based on its cuticle, composed of epicuticular and intracuticular waxes, which are essential for preventing water loss, minimising radiation-induced damage, combating pathogens, and coping with other stressors (Serrano et al., 2014). The gradual deposition of wax during ripening is crucial for preserving fruit quality after harvest, as it helps limit dehydration and reduce susceptibility to infection. For this reason, the integrity of the cuticle is essential to fruit preservation. Cuticular wax is mainly composed of triterpenoids and β-diketones, with ursolic acid and oleanolic acid being the main components. Fruit ripening leads to an increase in the levels of various wax constituents, such as alkanes, aldehydes, triterpenoids, fatty acids, and primary alcohols. On the other hand, the total wax content tends to decline during storage, although under low temperature and high relative humidity, there may be an increase in the total amount of triterpenoids (Yan and Castellarin, 2022), whose profile has a direct impact on water loss and fruit softening. In addition, higher levels of ursolic acid are associated with greater dehydration and lower firmness, whereas higher concentrations of oleanolic acid are associated with better water retention (Moggia et al., 2016; Kong et al., 2023). Therefore, selecting genotypes with an appropriate wax profile, including a higher oleanolic acid content and a lower proportion of wax esters that promote water loss, appears to be a good way to reduce dehydration and extend the shelf life of blueberries (Yan and Castellarin, 2022).

postharvest storage can influence the biological activity of phenolic compounds and anthocyanin levels, which are not entirely stable, as well as the fruit’s AC (Connor et al., 2002; Alvarez-Suarez et al., 2014). During storage, parameters such as pH, TSS, and TA may also change (Vieira et al., 2016; Liu et al., 2019; Rokayya et al., 2021), typically showing decreased TA and increased pH and TSS (Yan et al., 2020; Pratap-Singh et al., 2023). Enzymatic browning further reduces fruit lightness (L*) (Jiang et al., 2023).

### 4.1 Cold storage

There are several conservation strategies to extend the postharvest life of blueberries, and low-temperature storage remains the most widely used and effective technique, as it helps reduce water loss and maintains fruit firmness (Moggia et al., 2017b). This preservation system induces only moderate changes in pH, TSS, TA, texture, and fungal diversity (Yan et al., 2020; Wang et al., 2023b), and is clearly more effective than storage at higher temperatures, which accelerates sensory degradation, particularly aroma and flavour (Yan et al., 2020). Refrigeration and freezing can also promote the accumulation of phenolic compounds and anthocyanins, especially in late-harvested fruits (Mallik and Hamilton, 2017). Physiologically, cold temperatures delay ripening, preserve cell membrane integrity, and reduce lipid peroxidation, ROS production, and electrolyte leakage, thereby maintaining postharvest quality (Zhou et al., 2014). Thus, low temperatures close to 1–4 °C are essential to maintain quality and extend shelf life. In addition, storage at high relative humidity, between 90 and 95%, minimises weight loss and fruit shrivelling. Specifically, storing blueberries at 1 °C and 95% relative humidity has been shown to preserve their aromatic compounds and sensory attributes, whereas higher temperatures during transport and retail (10–20 °C) can negatively affect aroma and accelerate deterioration (Concha−Meyer et al., 2015; Cătuneanu et al., 2017; Kulapichitr et al., 2025). Although high relative humidity is crucial to prevent dehydration, it must be controlled to avoid microbial growth.

### 4.2 Controlled and modified atmosphere storage

In addition to conventional refrigerated storage, controlled atmosphere (CA) storage can modulate fruit biochemical composition. Applying CA conditions, such as 2.5 kPa of O_2_ and 15 kPa of CO_2_, for up to six weeks postharvest promoted an increase in the TPC of the ‘Centurion’ and ‘Maru’ cultivars. However, despite higher phenolic levels, antioxidant activity did not increase proportionally, indicating that responses depend on the preservation method (Eichholz et al., 2015). Similarly, CO_2_-rich atmospheres and/or SO_2_ fumigation helped maintain polyphenol levels and total antioxidant activity (Eichholz et al., 2015). The combination of CA, rapid cooling, and low temperatures has been shown to positively affect firmness, mass loss, TPC, and ascorbic acid content during storage (Kozos et al., 2014), though cultivar-specific variability remains significant (Maksimović et al., 2022). For extended storage, CA conditions with 5–12% CO_2_ and reduced O_2_ levels are commonly used (Forney, 2009; Cătuneanu et al., 2017).

### 4.3 Non-thermal and light-based treatments

Among non-thermal technologies, high-pressure processing (HPP), a commercial food pasteurisation technology, has proven effective in preventing mass loss and maintaining texture during cold storage. Specifically, HPP−treated blueberries can maintain firmness for up to 28 days without significant weight loss, although a slight reduction in instrumental firmness may occur immediately after processing (Scheidt and Silva, 2018). High hydrostatic pressure (400–600 MPa) may cause some cellular damage and lead to the release of bioactive compounds. However, AC is preserved, and texture is better maintained compared to blanching, partly attributed to the activity of cell wall-modifying enzymes, such as PME (Paciulli et al., 2019). Other non-thermal technologies have gained increasing attention for blueberry postharvest management. Cold plasma (CP) is a promising sanitisation approach that combines high antibacterial activity with the absence of chemical residues, making it particularly suitable for berries due to their delicate skin and irregular surfaces (Gonçalves et al., 2026). Beyond microbial control, CP has been reported to delay fruit softening by acting at the genetic level on physiological processes (Zhang and Cheng, 2024), inhibit native microbial growth and natural decay during storage (Hu et al., 2021), and preserve firmness, sugars, and ascorbic acid content. Notably, CP treatment has also been associated with elevated levels of total phenols, flavonoids, and anthocyanins, suggesting a stimulatory effect on phenolic metabolism (Zhou et al., 2023). Ozone treatment represents another viable non-thermal alternative, with pre-storage application shown to reduce fungal contamination on fruit surfaces without compromising quality. Continuous low-concentration ozone exposure further preserved anthocyanin content and maintained fruit appearance over time, supporting its potential as a practical strategy to extend the storage life of fresh blueberries (Takano et al., 2025). Compared with UV-C and aqueous ozone, CP demonstrated superior performance in inhibiting *Botrytis cinerea* growth and extending blueberry shelf life (Zhou et al., 2019). Light-based technologies have also been explored as complementary non-thermal approaches. The application of high-dose pulsed light has been shown to maintain fruit quality parameters such as firmness, weight loss and skin colour, thereby extending shelf life by up to six weeks, although accompanied by reduced AC (Pratap-Singh et al., 2023). Similarly, UV-B irradiation after harvest increased TPC and the levels of secondary metabolites, including terpenes, ketones, and C6 aldehydes, thereby improving the fruits’ bioactive properties (Eichholz et al., 2011).

### 4.4 Packaging

The adoption of innovative packaging can also help preserve blueberry quality during storage. Active packaging systems, such as polyethylene films incorporating salicylate nano−carriers combined with modified atmosphere packaging (MAP), can inhibit mould development and reduce respiration, extending shelf life by up to 13 days without affecting the sensory characteristics of the fruit (Bugatti et al., 2020). Biodegradable polylactide containers have also been shown to maintain better physicochemical and microbiological quality compared with conventional vented clamshells (Almenar et al., 2008, 2010). Furthermore, modified−humidity clamshells with controlled−release antifungal agents, such as thymol, reduce weight loss and decay (Olmedo et al., 2025), while humidity−responsive hydrogel packaging that releases antimicrobial essential oils offers additional potential for extending shelf life under high−humidity conditions (Shi et al., 2025).

### 4.5 Hormone-related and bioactive compound treatments

The use of physiological regulators and bioactive compounds further contributes to maintaining blueberry postharvest quality. Treatments involving endogenous plant regulators such as methyl jasmonate and ethanol vapour have been shown to slow fruit softening by inhibiting cell wall degradation and maintaining energy metabolism (Ji et al., 2021; Wang et al., 2021). The application of putrescine, a naturally occurring polyamine, also delayed softening by inhibiting the activity and expression of enzymes related to cell wall metabolism, thereby preserving fruit firmness (Song et al., 2022). Similarly, melatonin treatment contributed to quality preservation by reducing membrane lipid oxidation, the activity of cell wall-degrading enzymes, respiratory rates, and rot incidence, while preserving quality attributes such as TSS, TA, ascorbic acid, and fruit appearance after harvest (Qu et al., 2022).

### 4.6 Edible coatings

Edible coatings are thin, uniform films applied to food surfaces using one of three types of naturally occurring polymers: lipids, polysaccharides, or proteins. These coatings maintain the quality of fresh fruits and vegetables better than uncoated products by forming an environment around the coated food item that prevents moisture loss and gas exchange, reduces the rate of respiratory metabolism, provides a barrier to prevent contamination by bacteria and other microorganisms, and improves visual appeal (Kraśniewska et al., 2017; Ncama et al., 2018; Paidari et al., 2021; Matloob et al., 2023). Edible films containing hydrophobic materials can be formulated to enhance barrier properties when incorporated into the film as a topcoat or within the polymer matrix (Ghadermazi et al., 2019). Biopolymer-based edible coatings can be enriched with a variety of functional additives, including colorants, flavourings, probiotics and bioactive compounds with antioxidant and antimicrobial properties. These additives improve food quality and extend shelf life (Galus et al., 2020; Blancas-Benitez et al., 2022). The inclusion of such additives within the biopolymer matrix creates an adherent film that offers a sustainable alternative to conventional plastic packaging methods, providing an extended marketability, enhanced food safety and sensory qualities, and reduced food waste and costs (Sharma et al., 2019; Galus et al., 2020; Bizymis and Tzia, 2022). The effectiveness of edible films and coatings will largely depend on their physical, mechanical, and barrier properties. These properties are influenced by the chemical composition, compatibility, and miscibility of their components, particularly in multi-component systems (Galus et al., 2020). Therefore, it is important to comprehend the physiological behaviour and quality degradation of different types of fresh produce during postharvest storage to successfully develop and use edible coatings.

Coatings based on linear-structured polysaccharides, such as cellulose and its derivatives (e.g., pectin, chitosan, and gums), are strong, colourless, flexible, and highly soluble (Luangapai et al., 2019; Shao et al., 2020). There are several methods for applying edible coatings, including spreading, brushing, wrapping individually, and fluidized bed handling (Matloob et al., 2023). Compared with synthetic polymers, natural biopolymers offer several benefits; however, their high affinity with water limits their use and induces textural changes that significantly affect their solubility, mechanical properties, and transport characteristics (Cerqueira et al., 2012). To enhance or alter the material’s core properties, compounds such as emulsifiers, plasticizers, crosslinking agents, and reinforcements are added to film-forming formulations (Galus et al., 2020). The presence of plasticisers, such as glycerol, in films has been shown to modulate water vapour permeability and oxygen transmission rates depending on structural and physical properties of films, including solubility, opacity, mechanical characteristics, and water affinity. While plasticisers at optimal concentrations can increase film flexibility and elongation at break, excessive amounts may compromise tensile strength (Cerqueira et al., 2012; Ghadermazi et al., 2019). According to Skurtys et al. (2011), the highbush blueberry epicarp has a low surface free energy and is hydrophobic. However, removing the wax from the epicarp improves its wetting capacity, and blueberry epicarp is wetted more effectively by Tween 20/chitosan coating solutions than by glycerol/chitosan solutions.

The application of edible coatings has proven highly effective in preserving the quality of blueberries after harvest, as they act on various physiological and microbiological processes associated with the deterioration of this fruit (**Table 2**). Chitosan is among the most studied biopolymers, particularly for its versatility and antimicrobial activity. Applications of chitosan-based coatings have contributed to reducing the decay incidence, mass loss, and softening of blueberries from ‘Duke’, ‘Elliott’, ‘O’Neal’, and ‘Emerald’ cultivars, extending their shelf life (Duan et al., 2011; Chiabrando and Giacalone, 2015; Jiang et al., 2016; Hou et al., 2025). Fortification with aloe vera, blueberry leaf extract, essential oils (e.g., thyme, sunflower, carvacrol, cinnamaldehyde), dietary fibres, proteins, or nanomaterials can enhance these protective effects, ensuring better microbial control, firmness preservation, phenolic compounds, anthocyanins, and AC, while also extending the sensory attributes compared to the isolated application of chitosan (Sun et al., 2014; Yang et al., 2014; Abugoch et al., 2016; Vieira et al., 2016; Alvarez et al., 2018; Rokayya et al., 2021). Advanced chitosan formulations, through the use of nanoparticles, nanostructured films, or combinations with physical treatments (e.g., UV-C, thermal shock), have yielded promising outcomes in maintaining cell wall integrity, the reduction of gene expression associated with polysaccharide degradation, and preventing softening during blueberry storage (Sun et al., 2022; Huang et al., 2023; Liu et al., 2023; Correa-Pacheco et al., 2025). A decrease in microbial populations and structural collapse rates was further observed after the application of coatings combining chitosan with silicon dioxide, titanium dioxide, nisin, or natural waxes (Eldib et al., 2020; Li et al., 2021a; Mohamed and Ramaswamy, 2025).

**Table 2:** Summary of edible coating formulations applied postharvest to blueberries and their effects on quality and shelf-life parameters.

Coating/film composition	Effect on blueberries	Method of application	References
Chitosan	Delayed softening and senescence; increased firmness; reduced weight loss, decay, and microbial load; modulation of titratable acidity (TA), total phenolic content (TPC), anthocyanins, and antioxidant capacity (AC); improved sensory quality (appearance, texture, flavour)	Dipping	Sun et al. (2014); Chiabrando and Giacalone (2015); Jiang et al. (2016); Alvarez et al. (2018)
CMC/chitosan layer-by-layer (LBL) coatings	Extended shelf life under ambient conditions; reduced rotting and weight loss rates; better maintenance of firmness, TSS, respiration rate, and AC compared with single-layer coatings	Dipping	Hou et al. (2025)
Chitosan nanoparticles (NP) into chitosan/polyvinyl alcohol (PVA) coatings	Reduced weight loss and decay; maintained firmness; increased anthocyanin content during storage; strong antifungal activity against *Botrytis cinerea*; improved structural, thermal, and mechanical film properties	Spraying	Correa-Pacheco et al. (2025)
Chitosan + thyme essential oil (± UV-C)	Strong inhibition of postharvest softening and senescence via suppression of cell wall-degrading enzymes and related gene expression; maintained cell wall polysaccharides; combined coating + UV-C showed the greatest effect	Dipping	Sun et al. (2022)
Chitosan + blueberry leaf extract (BLE)	Increased firmness, TPC, and AC; reduced weight loss and decay; delayed softening; synergistic effects observed under modified atmosphere packaging (MAP)	Dipping; cold storage ± MAP	Yang et al. (2014)
Chitosan + essential oils (carvacrol, trans-cinnamaldehyde)	Reduced microbial growth and decay incidence; improved firmness during storage	Dipping	Sun et al. (2014)
Chitosan + aloe vera	Delayed quality deterioration; maintained firmness and titratable acidity; extended shelf life	Dipping	Vieira et al. (2016)
Chitosan + nano-SiO_2_/nano-TiO_2_ (± nisin)	Improved textural properties; reduced weight loss, decay, shrinkage, and membrane damage; maintained anthocyanins and vitamin C; reduced microbial populations; delayed ripening processes	Dipping	Eldib et al. (2020); Li et al. (2021b); Rokayya et al. (2021)
Chitosan + sodium alginate	Increased titratable acidity, TPC, anthocyanins, and AC; reduced mould growth	Dipping	Chiabrando and Giacalone (2015)
Konjac glucomannan + low-acyl gellan gum (KGM/LAG)	Reduced weight loss and decay; lowered respiration rate, lipid peroxidation, and membrane leakage; increased TPC; improved storage stability	Spraying	Huang et al. (2023); Liu et al. (2023)
KGM/LAG + thymol microcapsules or nanoparticles	Strong delay of postharvest softening through modulation of cell wall disassembly; reduced activity of cell wall-degrading enzymes; altered pectin, hemicellulose, and cellulose fractions; reduced oxidative damage and respiration rate	Preharvest spraying	Huang et al. (2023); Ding et al. (2024)
Pullulan	Reduced weight loss and decay; increased fructose, glucose, and vitamin C contents during storage	Dipping	Kraśniewska et al. (2017)
Pullulan ± propolis extract	Delayed weight loss; reduced bacterial and mould counts; modulation of titratable acidity	Brushing	Pobiega et al. (2021)
Gum arabic (± roselle or baobab extracts)	Reduced weight loss and decay; increased firmness, TPC, anthocyanins, and AC; reduced enzymatic browning and microbial growth; improved overall postharvest quality	Dipping	Yang et al. (2019); Tahir et al. (2020)
Sodium alginate (± CaCl_2_)	Maintained firmness; increased TPC, anthocyanins, and AC; reduced decay and microbial populations; improved phenolic stability	Dipping	Chiabrando and Giacalone (2015); Mannozzi et al. (2017)
Sodium alginate + carvacrol	Reduced weight loss, respiration rate, and decay; improved firmness, colour (L*), TPC, and shelf life via antimicrobial activity	Dipping + calcium gelling bath	Medina-Jaramillo et al. (2020)
Sodium alginate (± inulin, fruit fibre or oligofrutose)	Reduced decay; increased firmness, TPC, and AC	Dipping	Alvarez et al. (2018)
Pectin ± sodium alginate	Increased firmness; reduced mesophilic bacteria and yeasts	Double dipping	Mannozzi et al. (2017)
Carboxymethyl cellulose (CMC)	Reduced decay and water loss; maintained firmness; increased TPC and vitamin C; reduced lipoxygenase activity; enhanced AC	Dipping	Totad et al. (2019); Totad et al. (2020)
CMC ± propolis extract	Reduced weight loss; increased TPC and AC; reduced yeasts and fungi; improved microbial stability	Dipping	Tumbarski et al. (2022)
CMC + beeswax composites	Reduced moisture loss and respiration rate; delayed quality deterioration during cold storage	Double dipping	Rodríguez-Nieto et al. (2019)
Quinoa protein + chitosan + sunflower oil	Reduced pH and microbial growth; increased titratable acidity; improved shelf life	Dipping	Abugoch et al. (2016)
Calcium caseinate	Improved firmness and maintenance of postharvest quality under commercial storage conditions	Double dipping	Duan et al. (2011)
Xanthan gum/guar gum/gum arabic	Reduced physiological weight loss and decay; increased firmness; reduced lipoxygenase activity	Dipping	Totad et al. (2019); Totad et al. (2020)
Citrus pectin-based coatings (CP, PPC, PPN)	Reduced murine norovirus (MNV) and hepatitis A virus (HAV) contamination on fruit surface	Dipping	Méndez et al. (2023)
Caseinate–carboxymethyl chitosan composite + soybean oil	Reduced weight loss and microbial growth; better retention of firmness and pH; extended shelf life compared with uncoated fruit	Dipping	Mohamed and Ramaswamy (2025)
*Chlorella vulgaris* protein ± rosemary extract	Reduced weight loss; increased TPC and AC; preserved bioactive profile; reduced microbial counts; extended shelf life	Dipping	Mari et al. (2025)
*Hanseniaspora uvarum* + konjac glucomannan	Reduced weight loss and postharvest diseases; improved firmness, colour (L*), and antioxidant enzyme activity	Spraying	Jiang et al. (2023)

AC, antioxidant capacity; TA, titratable acidity; BLE, blueberry leaf extract; CMC, carboxymethyl cellulose; KGM, konjac glucomannan; LAG, low-acyl gellan gum; LBL, layer-by-layer; MAP, modified atmosphere packaging; NP, nanoparticles; PVA, polyvinyl alcohol; TPC, total phenolic content; TSS, total soluble solids.

Beyond chitosan, other natural polymers, such as alginate, pectin, carboxymethylcellulose (CMC), natural gums, plant or animal proteins, and emerging polysaccharides (e.g., pullulan and konjac glucomannan), have shown great potential for blueberry preservation. Numerous studies have shown that firmness, TA, and colour can be maintained when alginate-based coatings are used alone or synergistically with pectin, dietary fibres, or antimicrobial agents, while reducing microbial load across various blueberry cultivars and prolonging shelf life for as long as 21 days during cold storage (Chiabrando and Giacalone, 2015; Mannozzi et al., 2017; Alvarez et al., 2018; Medina-Jaramillo et al., 2020). Similarly, CMC and polymer mixtures also contributed to reduced transpiration, delayed ripening, conserved bioactive compounds, and improved sensory attributes, enabling storage periods of 18 to 35 days (Totad et al., 2019, 2020; Rodríguez-Nieto et al., 2019; Tumbarski et al., 2022).

**Figure 2:**
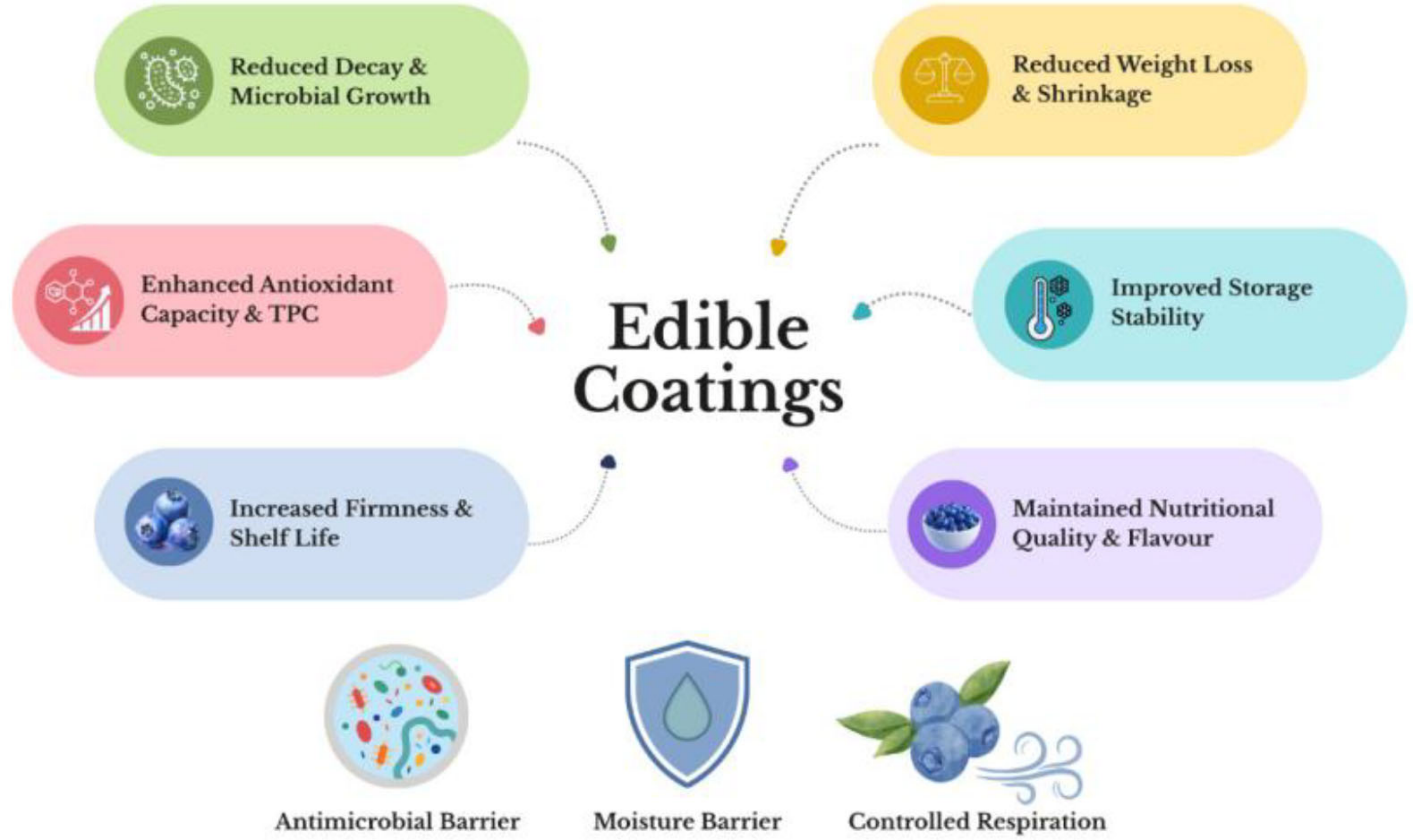
Benefits of edible coatings for blueberry fruit preservation.

The use of coating materials produced from konjac glucomannan combined with gellan gum hydrogels, particularly when enriched with thymol nanoparticles, increases blueberry storage life up to 42 days at lower temperatures, as a result of lipid peroxidation reduction, epidermal integrity maintenance and enhancements to antioxidant defence enzyme activity (Huang et al., 2023; Jiang et al., 2023; Liu et al., 2023; Ding et al., 2024). Gum Arabic coatings, whether alone or supplemented with extracts from roselle and/or baobab, have been shown to reduce weight loss and spoilage while elevating firmness, phenolics, AC, and suppressing enzymatic browning and microbial growth (Yang et al., 2019; Tahir et al., 2020). Additional complementary strategies incorporating pullulan, melatonin, microalgal proteins, or combinations with osmotic dehydration in blueberries, promoted moisture retention, antioxidant stability, and microbial control (Kraśniewska et al., 2017; Pobiega et al., 2021; Li et al., 2024; Mari et al., 2025). This demonstrates the potential and effectiveness of edible coatings as sustainable tools for postharvest preservation of blueberries (**Figure 2**).

## 5 Future perspectives

The continued improvement of blueberry fruit quality will increasingly depend on the integration of emerging scientific tools with sustainable agronomic practices. High-throughput omics technologies represent a particularly transformative opportunity in this regard. Transcriptomic and metabolomic analyses have already proven valuable for dissecting the regulatory networks underlying important quality traits, including anthocyanin accumulation, sugar-acid balance, and antioxidant capacity (Yang et al., 2023; Chu et al., 2024). Extending these approaches to proteomics holds considerable promise for identifying robust molecular markers applicable to precision breeding and non-destructive cultivar selection across diverse growing environments.

At the genetic level, CRISPR/Cas9 gene editing offers unprecedented precision for targeted improvement of highbush blueberry cultivars. Combined with genome-wide association studies and transcriptomic data, this technology enables the rational modification of traits such as phenylpropanoid metabolism, cuticular wax biosynthesis, and pathogen resistance (Vaia et al., 2022; Gonçalves et al., 2026), potentially accelerating the development of cultivars with superior performance after harvest without the limitations of conventional breeding timescales.

From an agronomic perspective, the standardisation and regulatory recognition of biostimulant products remains a priority. Realising the full potential of biostimulants will require transdisciplinary research that bridges agronomy, environmental science, and food technology, alongside robust policy frameworks, certification schemes, and farmer training programmes to support their broader adoption within ecological intensification strategies (Gonçalves et al., 2025).

Finally, the integration of pre- and postharvest strategies into commercially viable systems represents perhaps the most immediate opportunity for impact. The combination of preharvest biostimulant application with postharvest preservation technologies may offer promising synergistic effects on productivity, shelf life, and nutritional quality (Gonçalves et al., 2026), yet systematic evaluation under real commercial conditions remains limited. Closing this gap through coordinated multisite trials that account for cultivar, environment, and supply chain context should be a central priority for the field in the next decade.

## 6 Conclusions

The quality of blueberries, defined by their physical attributes (biometrics, firmness, colour, and flavour), as well as their biochemical composition and antioxidant capacity, is influenced by genetic and environmental factors, and cultural practices. Given that blueberry quality at harvest influences its shelf life, growers must find the optimal balance between the vegetative and reproductive growth, ensure adequate water and nutrient availability, and harvest at the optimal maturity stage. By improving plant physiological vigour and efficiency, preharvest application of biostimulants can enhance fruit quality. Calcium is essential for cell wall stabilization, preserving fruit firmness. However, the effectiveness of these treatments depends on formulation, dosage, timing, application method, and fruit absorption capacity during development. postharvest storage potential of blueberries depends mainly on refrigerated storage. Alternative technologies have become available, including edible coatings made from biodegradable polymers. Particularly when functionalized with antimicrobial/antioxidant agents, these coatings extend marketability and preserve commercial characteristics while reducing quality loss from dehydration and decay. Knowledge gaps persist in terms of development and adoption of standardized research protocols and in comparing study outcomes, as well as in understanding how various blueberry cultivars respond to different treatment methods. To provide growers with the resources and knowledge needed to optimize the financial and nutritional returns from their crops while ensuring the long-term sustainability of the production system, long-term, multi-year field studies evaluating yield, organoleptic properties, and postharvest quality are required.
